# The European Medical Device Ecosystem: Interactions Between Regulation, Innovation, Market Access, and Healthcare Systems

**DOI:** 10.3390/healthcare14213822

**Published:** 2026-11-09

**Authors:** Bagdat Azamatov, Marzhan Sadenova, Stanislav Boldyryev

**Affiliations:** 1 Center of Excellence «VERITAS», D. Serikbayev East Kazakhstan Technical University, Ust-Kamenogorsk 070000, Kazakhstan; bazamatov@edu.ektu.kz (B.A.); msadenova@edu.ektu.kz (M.S.); 2 Faculty of Mechanical Engineering and Naval Architecture, The University of Zagreb, 10000 Zagreb, Croatia

**Keywords:** medical devices, medical device regulation, health technology assessment, market assessment, regulation–innovation interaction, healthcare systems

## Abstract

The European Union medical device sector is analysed in several largely separate strands of the literature: regulatory studies of the Medical Device Regulation (MDR) and the In Vitro Diagnostic Regulation (IVDR), innovation research on individual technology classes, commercial market reporting, and health-policy work on adoption and reimbursement. Integration across these dimensions is limited, so the mechanisms by which regulatory change propagates into innovation, market access and healthcare-system outcomes remain poorly specified. This review addresses that gap by proposing and applying a five-stage conceptual framework: Regulation, Evidence, Innovation, Market Access, and Healthcare-System Impact, and by using it to structure a reproducible literature synthesis. Four bibliographic databases and a defined set of institutional sources were searched for the period January 2017 to June 2026, with a targeted update to October 2026 and the geographical scope restricted to the EU-27 and the European Economic Area (EEA); identified records were screened against explicit criteria, and the retained documents were coded against the five framework dimensions. Search, screening and coding records are reported in full, with the PRISMA flow diagram, checklist, screening log and coded corpus provided. Rather than reporting market values as isolated figures, the synthesis examines relationships between certification duration, evidence intensity, segment growth, market concentration and access conditions. Three findings emerge. First, at the segment level, the association between evidence intensity and growth runs in the opposite direction to the one usually assumed: the segments with the heaviest evidence burden, Class III implantable and Class C/D in vitro diagnostics, grow in the mid-single digits, whereas the fastest-growing segments, digital health and wearables, sit predominantly in lower conformity-assessment classes. Certification duration is nevertheless substantial throughout, with median application-to-certificate totals reported at approximately 17–19 months for in vitro diagnostics and 19–22 months for medical devices, so that evidence requirements act less as a uniform brake on growth than as a selection mechanism determining which firms can participate in the most regulated segments. Second, the innovation consequence of regulatory delay has moved from assertion to estimation: a two-market economic model estimates that introducing a three-year supplementary protection certificate limited to Class III devices would raise EU-directed development effort by 13.46% in a single-market EU calibration, and by 5.6% in the two-market setting and manufacturer survey evidence records a reallocation of first approvals and launches away from the EU. Third, the December 2025 targeted revision of the medical device regulation (MDR) and in vitro diagnostic regulation (IVDR), implementing regulation (EU) 2026/977 setting maximum conformity-assessment timelines, the selection of the first two medical devices for joint clinical assessment in October 2026, and the phased mandatory use of the European database on medical devices (EUDAMED) together constitute a partial correction whose effects are not yet observable. The framework identifies where regulatory change translates into consequences for manufacturers, providers and patients, and shows that the chain is well evidenced at its regulatory origin and weakest at its healthcare-system terminus.

## 1. Introduction

The European medical instruments production sector serves as a vital foundation of the continent’s healthcare system and represents a market of considerable global importance. With an anticipated valuation exceeding €170 billion in 2024, Europe accounts for approximately one-quarter of the worldwide MedTech demand [1]. Key manufacturing and export centres, including Germany, Ireland, the Netherlands, and Italy, underpin Europe’s industrial landscape and contribute to a modest yet steady trade surplus. Demographic trends, notably population ageing and the increasing prevalence of chronic diseases, are fuelling demand for diagnostic, surgical, and therapeutic instruments [2]. Concurrently, reforms within healthcare systems and the pivot towards outpatient and home-based care are altering consumption patterns, paving the way for opportunities in minimally invasive, digital, and connected technologies [3].

The regulatory landscape has been significantly altered by the introduction of the MDR [4] and the IVDR [5]. While these frameworks enhance patient safety and product quality, they have also led to increased compliance costs, longer approval timelines, and heightened pressure on notified bodies’ capacity. Small and medium-sized enterprises encounter unique hurdles in this environment, although the elevated standards can create competitive advantages for those well equipped to meet them. Technological innovation continues to be the primary driver of growth in the sector [6]. Developments in AI-driven diagnostics [7], robotics [8], wearable health monitoring [9], molecular point-of-care testing [10], and telemedicine integration is transforming the competitive landscape. Although established areas such as imaging, orthopaedics, and disposable medical products are expanding at a steady pace, the most rapid growth is being observed in digital health and advanced surgical systems [11].

Four distinct bodies of literature address this sector, and they rarely meet. The first is regulatory: studies of the MDR and IVDR examine legal scope, conformity-assessment procedure, notified-body capacity and transitional relief, and evaluate the framework largely against its own stated objective of patient safety. The second is technological: research on artificial-intelligence-enabled diagnostics, surgical robotics, wearable monitoring, molecular point-of-care testing and additive manufacturing characterises device capability and clinical performance, usually without reference to the regulatory pathway that determines whether and when those capabilities reach patients. The third is commercial: industry and consultancy reporting quantifies market size, segment growth and merger activity, but treats regulation as an exogenous cost shock rather than as a variable interacting with the others. The fourth is health-policy: work on health technology assessment, procurement and reimbursement analyses adoption and access conditions, generally taking the supply of certified devices as given.

Each literature is internally coherent, and each is incomplete in the same way. Regulatory analysis does not quantify innovation consequences; innovation research does not model market access; market reporting does not explain the mechanism behind the growth rates it reports; and health-policy research does not trace access conditions back to conformity assessment. What is missing is an integrated synthesis explaining how regulatory transformation interacts with innovation, market access and healthcare-system consequences across the European Union. That gap is the subject of this review.

The gap matters now because the European framework is not static. On 16 December 2025, the European Commission published COM (2025) 1023, a targeted revision of both Regulations intended to reduce procedural burden, introduce statutory conformity-assessment timelines, create priority and rolling-review pathways for breakthrough and orphan devices, and clarify the interaction with the Artificial Intelligence Act [12]. The proposal is a response to the Commission’s own targeted evaluation, which found conformity assessment to be overly complex and costly, legal requirements to be inconsistently applied across Member States, and the Regulations to have had unintended negative effects on competitiveness and on patient care [13]. In parallel, the Health Technology Assessment Regulation has moved from medicines to devices, with the first joint clinical assessments of high-risk devices scheduled to begin in mid-2026 [14], and the EUDAMED transparency obligations became applicable on 28 May 2026 [15]. At the same time, the empirical literature has begun to quantify the innovation cost of regulatory delay, through an explicit two-market economic model of EU and US frameworks [16] and through survey evidence reporting a reallocation of first approvals and launches outside the EU [17]. Three further developments fall within the revised search window. Commission Implementing Regulation (EU) 2026/977, published on 5 May 2026 and applicable from 25 February 2027, lays down binding maximum timelines for each phase of conformity assessment and obliges notified bodies to monitor and publish the duration and cost of their assessments [18]. The Digital Omnibus, which the Council gave final approval on 29 June 2026, left the MDR and the IVDR in Section A of Annex I to the Artificial Intelligence Act, keeping AI-enabled devices as high-risk AI systems while deferring the corresponding obligations to 2 August 2028 [19]. And on 6 October 2026, the Commission selected the first two medical devices for joint clinical assessment [20]. A review that treats regulation, innovation and access separately cannot accommodate these findings; a framework that links them can.

This review therefore proposes a five-stage conceptual framework: Regulation, Evidence, Innovation, Market Access, and Healthcare-System Impact, and uses it both as an analytical device and as the classification scheme for a structured literature synthesis. The framework treats regulation not as a terminal constraint but as the first node in a causal chain: regulatory requirements determine the evidence a manufacturer must generate; evidence requirements determine the cost, duration and risk of development, and therefore the level and the location of innovation; innovation outcomes, filtered through health technology assessment and procurement, determine market access; and market access determines what healthcare systems can actually deploy. Four questions follow: (i) what has changed in the EU regulatory framework, and why; (ii) what measurable effects does that change have on evidence generation, certification duration and compliance cost; (iii) how do those effects propagate into innovation behaviour and market structure; and (iv) what are the observable and prospective consequences for manufacturers, healthcare providers and patients?

The contribution is threefold. First, the review integrates four bodies of literature that have developed independently and specifies the transmission mechanisms between them rather than asserting that they are related. Second, it applies a transparent and reproducible search and classification protocol, set out in Section 2, so that the synthesis can be audited and updated as the 2025–2026 reforms take effect. Third, it moves the quantitative treatment of the European market from the reporting of isolated values to the examination of relationships between variables, certification duration, evidence intensity, segment growth and market concentration, and grades the evidence behind each relationship. The remainder of the paper follows the framework: Section 2 sets out the framework and the review methodology; Section 3 establishes the market baseline; Section 4 and Section 5 address the regulatory and evidence nodes; Section 6, Section 7 and Section 8 address demand drivers, compliance burden and competitive selection; Section 9 and Section 10 address innovation and external market access; Section 11 presents the quantitative synthesis of relationships; Section 12 discusses healthcare-system implications, limitations and a research agenda; and Section 13 concludes.

## 2. Conceptual Framework and Review Methodology

### 2.1. An Integrated Framework Linking Regulation, Evidence, Innovation, Market Access and Healthcare-System Impact

The framework adopted here treats the European medical device ecosystem as a directed chain of five dimensions rather than as a set of parallel topics. Regulation defines what must be demonstrated before a device may be placed on the market: risk classification, conformity-assessment route, clinical evaluation requirements, post-market obligations and traceability. Evidence responds to that demand: the clinical investigations, performance studies, clinical evaluation reports, surveillance systems, and real-world data a manufacturer must produce, along with the time and capital required. Innovation is conditioned by that evidence burden, because the expected cost, duration and probability of certification enter directly into the decision to develop a device, to develop it for one jurisdiction before another or not to develop it at all. Market access is a second filter: certification permits sale but does not secure adoption, which depends on health technology assessment, reimbursement and procurement. Healthcare-system impact is the terminal dimension: the availability, cost and clinical value of the devices that actually reach providers and patients.

The chain is not unidirectional. Post-market evidence generated under the MDR and IVDR feeds back into classification and vigilance; observed shortages and withdrawals feed back into legislative revision, as the December 2025 proposal demonstrates; and health technology assessment requirements increasingly shape the evidence manufacturers plan before certification rather than after it, which moves the market-access filter upstream into the evidence dimension. Two feedback loops are therefore treated explicitly: a surveillance loop running from healthcare-system impact back to regulation and an anticipation loop running from market access back to evidence design.

The analytical value of the framework is that it converts descriptive observations into testable relationships. Statements such as “the MDR increased compliance costs” or “digital health is growing quickly” become, within the framework, claims about transmission: how large an effect, transmitted through which node, observable in which data, and with what consequence at the next node. Where a link in the chain is supported by quantitative evidence, this review reports the estimate and its identification strategy; where the link is asserted but not measured, the review says so. Section 11 collects and grades these links, and Section 12 identifies those that remain unevidenced.

### 2.2. Review Methodology

This review is a structured, protocol-driven synthesis rather than a systematic review with meta-analysis: the outcome measures in the underlying literature, certification duration, compliance cost, market value, adoption rate, and development effort are too heterogeneous in definition and provenance to be pooled statistically. The identification, screening and reporting steps nonetheless follow and are reported against the PRISMA 2020 reporting principles [21] so that the synthesis is reproducible and its boundaries are explicit. The completed PRISMA 2020 checklist is provided as Supplementary Material S1, the flow diagram as Supplementary Material S2, the full list of included documents with their codes as Supplementary Material S3, and the screening log as Supplementary Material S4.

• *Databases searched*. Scopus, Web of Science Core Collection, PubMed/MEDLINE and ScienceDirect. Institutional and official sources were searched separately and by hand: EUR-Lex; the European Commission Directorate-General for Health and Food Safety (DG SANTE) medical devices and health technology assessment pages; the Medical Device Coordination Group (MDCG) guidance repository; EUDAMED documentation; Eurostat; the OECD; and MedTech Europe publications.
• *Search period*. Records dated 1 January 2017 to 30 June 2026, with institutional and legal sources updated to 10 October 2026 during revision to capture Implementing Regulation (EU) 2026/977, the Digital Omnibus, and the first selection of devices for joint clinical assessment. The start date is the adoption of Regulations (EU) 2017/745 and (EU) 2017/746. Legal instruments predating 2017 were consulted only where required to establish the counterfactual represented by the Directives.
• *Search strings*. Applied to title, abstract and keywords and adapted to each database’s syntax: (i) (“medical device*” OR “in vitro diagnostic*”) AND (“Medical Device Regulation” OR MDR OR IVDR OR “2017/745” OR “2017/746”) AND (Europe* OR “European Union” OR EU); (ii) (“medical device*”) AND (innovation OR “R&D” OR “product development”) AND (regulat* OR certification OR “conformity assessment”) AND (Europe* OR EU); (iii) (“medical device*” OR “health technolog*”) AND (“health technology assessment” OR HTA OR reimbursement OR procurement OR “market access”) AND (Europe* OR EU); (iv) (“medical device*”) AND (“notified body” OR “clinical evidence” OR “post-market surveillance” OR EUDAMED OR “unique device identification”); (v) (“medical device*”) AND (market OR “market size” OR Compound Annual Growth Rate (CAGR) OR trade OR “supply chain”) AND (Europe* OR EU).
• *Inclusion criteria*. (a) The record addresses medical devices or in vitro diagnostic medical devices as defined in Regulations (EU) 2017/745 and (EU) 2017/746; (b) it concerns the EU or EEA regulatory, innovation, market-access or healthcare-system context, or provides an explicit EU comparison; (c) it reports data, empirical evidence, legal analysis or official policy rather than opinion alone; (d) it is published in English; (e) it falls within the search period.
• *Exclusion criteria*. (a) Medicinal products, and combination products assessed principally as medicines; (b) purely clinical studies of a single device with no regulatory, economic or system-level dimension; (c) jurisdictions outside the EU/EEA, except where used as an explicit comparator; (d) vendor marketing material, press releases and commentary without a verifiable data source; (e) duplicate reporting of the same underlying dataset, in which case the most complete version was retained; (f) records for which only an abstract was retrievable.
• *Geographical definition of the EU*. The EU-27, together with the EEA States (Iceland, Liechtenstein, Norway) to which the Regulations apply. Switzerland and the United Kingdom are treated as third countries and are included only where they bear on EU market access or on the measurement of mutual recognition and Brexit effects. United States material is included only for comparative regulatory economics.
• *Document types*. Peer-reviewed research articles and reviews; EU primary legislation and legislative proposals; regulatory guidance (MDCG) and competent-authority documents; official statistics (Eurostat, OECD); trade-association surveys and position papers; and commercial market reports. Commercial reports are used only to triangulate orders of magnitude, are never the sole source for a quantitative claim, and are always reported as a range spanning at least two independent providers.
• *Screening process*. Records were de-duplicated, screened on title and abstract against the inclusion criteria, and then assessed in full text. Screening was performed independently by two authors, with disagreements resolved by discussion with the third; legal and institutional sources were verified directly against the issuing body’s published text rather than through secondary reporting.
• *Records identified and retained*. Database searching identified 6226 records (Scopus 2434, Web of Science 1310, PubMed/MEDLINE 1459, ScienceDirect 1023) and institutional or hand searching identified a further 500 documents. After removal of 4729 duplicates, 1997 records were screened by title and abstract, of which 1706 were excluded. 291 full texts were assessed for eligibility, and 198 were excluded, with reasons recorded in Supplementary Material S2 (28 no EU/EEA dimension, 33 single-device clinical study, 41 no verifiable data source, 25 duplicate dataset, 33 abstract only). 93 documents were retained for synthesis, comprising 41 peer-reviewed publications, 5 legislative and regulatory documents, 17 regulatory guidance documents, 6 statistical publications, 19 market or trade-association reports, and 5 company and research reports. The corresponding PRISMA flow diagram is provided as Supplementary Material S2, and the complete screening log, including all excluded records and exclusion reasons, is provided as Supplementary Materials S3 and S4.

Each retained document was then coded against a three-axis classification framework. The first axis records the framework dimension addressed: Regulation, Evidence, Innovation, Market Access, and Healthcare-System Impact, with multiple codes permitted and a cross-cutting code applied when a document explicitly links two or more dimensions. The second axis records evidence type: quantitative-empirical, qualitative-empirical, modelling or economic analysis, legal or policy analysis, and descriptive or market reporting. The third axis records device domain: in vitro diagnostics; diagnostic imaging; implantable devices; surgical instruments and robotics; software as a medical device and digital health; single-use and consumable devices; and cross-domain. The coding was performed independently by two authors on the full retained corpus; inter-rater agreement and the full coded dataset are reported in Supplementary Material S3.

### 2.3. Treatment of Quantitative Material

Quantitative material is handled under four rules. First, any market value is reported as a range spanning at least two independent sources, and the scope of each estimate—devices only, devices with IVD reagents, devices with associated services—is stated where the sources disclose it, because differences in scope account for much of the apparent disagreement between providers. Second, currency conversion uses the 2024 average EUR/USD rate of 1.0822, and the sensitivity of the resulting figures to that choice is acknowledged rather than suppressed. Third, growth projections are presented as explicit scenarios rather than as point forecasts, with the scenario assumptions stated alongside the figure. Fourth, only one indicator is constructed by the authors: the segment evidence-intensity classification set out in Table 1, which assigns each market segment to the predominant risk class and conformity-assessment route prescribed by Annex VIII of regulations MDR and IVDR [19]. It is a rule-based mapping from published legal classification criteria, not a weighted composite score, and its derivation is stated in full in Table 1 so that it can be reproduced or contested. The import-dependency and tender-risk illustrations presented in the submitted version rested on synthetic country-level estimates for which no underlying data could be documented; they have been withdrawn in this revision, together with the associated figures and text. No causal estimate reported in this review was generated by the authors; causal claims are attributed to the primary studies that produced them, together with their identification strategy and its limitations.

**Table 1:** Evidence-intensity classification of market segments, derived from the classification rules of Annex VIII of Regulation (EU) 2017/745 and Regulation (EU) 2017/746. The classification is rule-based and records the predominant case within each segment; it is not a weighted index.

Segment	Predominant Risk Class	Conformity-Assessment Route	Evidence intensity
Single-use and consumable devices	MDR Class I/IIa	Self-declaration or limited notified-body involvement	Low
Diagnostic imaging (replacement capacity)	MDR Class IIa/IIb	Notified body; technical documentation assessment	Moderate
Surgical instruments (non-powered)	MDR Class I/IIa	Self-declaration or limited notified-body involvement	Low
Digital health, wearables and software without a diagnostic claim	Not a device, or MDR Class I	Outside the Regulations or self-declaration	Low
Software as a medical device with a diagnostic or therapeutic claim	MDR Class IIa/IIb (Rule 11)	Notified body; AI Act obligations in addition	Moderate to high
In vitro diagnostics, general	IVDR Class B/C	Notified body for Class B and above	Moderate
Molecular and companion in vitro diagnostics	IVDR Class C/D	Notified body; EU reference laboratory for Class D	High
Surgical robotics	MDR Class IIb	Notified body; clinical investigation usual	High
Implantable devices (orthopaedic, cardiovascular)	MDR Class III/implantable IIb	Notified body; clinical investigation and expert-panel consultation	Highest

## 3. Market Structure and Growth: The Demand Baseline

**Figure 1:**
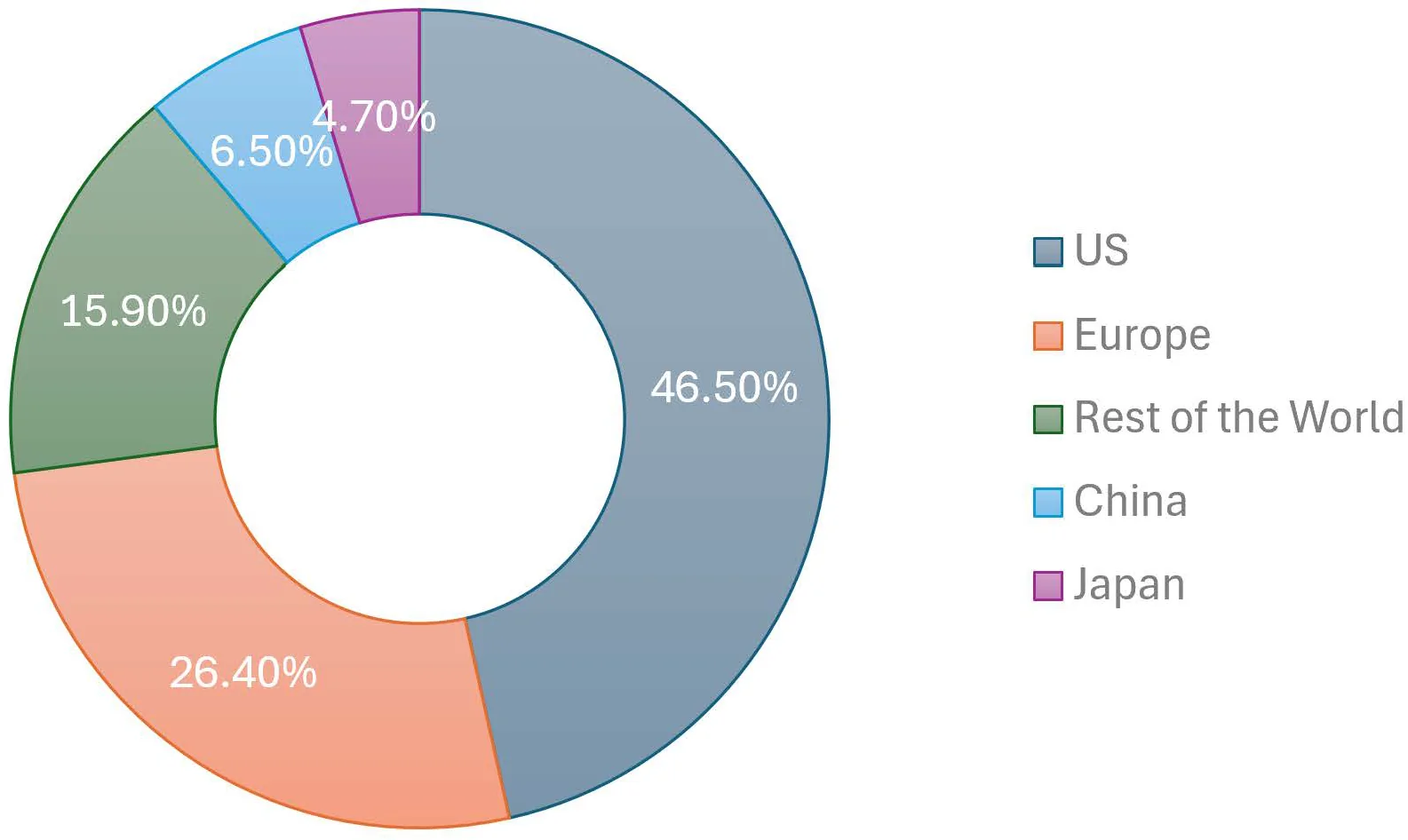
Global medical device market by region in 2024. Shares derived from the market-size estimates reported in [22,23,25]. Produced in Python 3.11 (Matplotlib 3.8).

The EU medical instruments sector is one of the world’s largest, valued at €131–170 billion in 2024 [22,23]. Market size estimates vary by scope, but all sources agree on steady growth, projected at 4–6% CAGR through 2030 [24]. Key drivers include an ageing population, rising demand for diagnostics and long-term care, advances in minimally invasive and AI-powered technologies, and rapid adoption of digital health. Even conservative forecasts predict the market will add tens of billions in value within the next decade. The world medical device market by region for 2024 is demonstrated in Figure 1.

**Figure 2:**
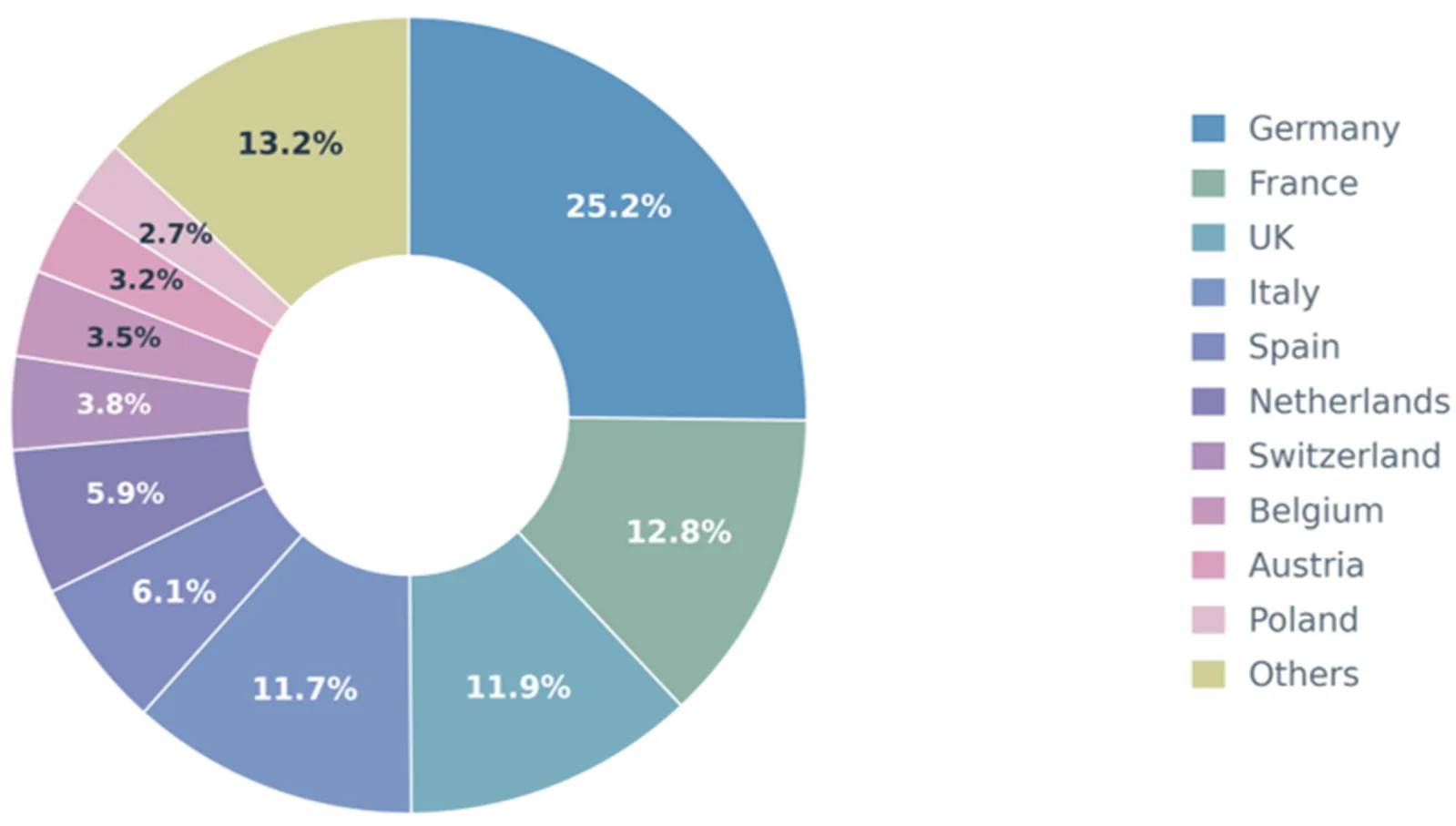
European medical device market by country in 2024, as a share of the geographic European market. Values from [26,27]; shares below 3% are grouped as “Other”. The United Kingdom and Switzerland are included because the source data report geographic Europe; they are outside the EU/EEA regulatory scope defined in Section 2.2. Produced in Python 3.11 (Matplotlib 3.8).

The European market is also highly concentrated in a handful of national economies, and the share of the medical device market by country is demonstrated in Figure 2. Germany remains the clear leader, representing roughly a quarter of the total market, with estimates of around €38–43 billion in 2024 [26]. France, Italy and Spain follow as the next largest EU markets; the United Kingdom, although no longer a Member State, is shown for comparability with the source data, which report a geographic rather than a regulatory Europe. Together, these markets account for a further 35–40% of European medical device consumption [27].

**Figure 3:**
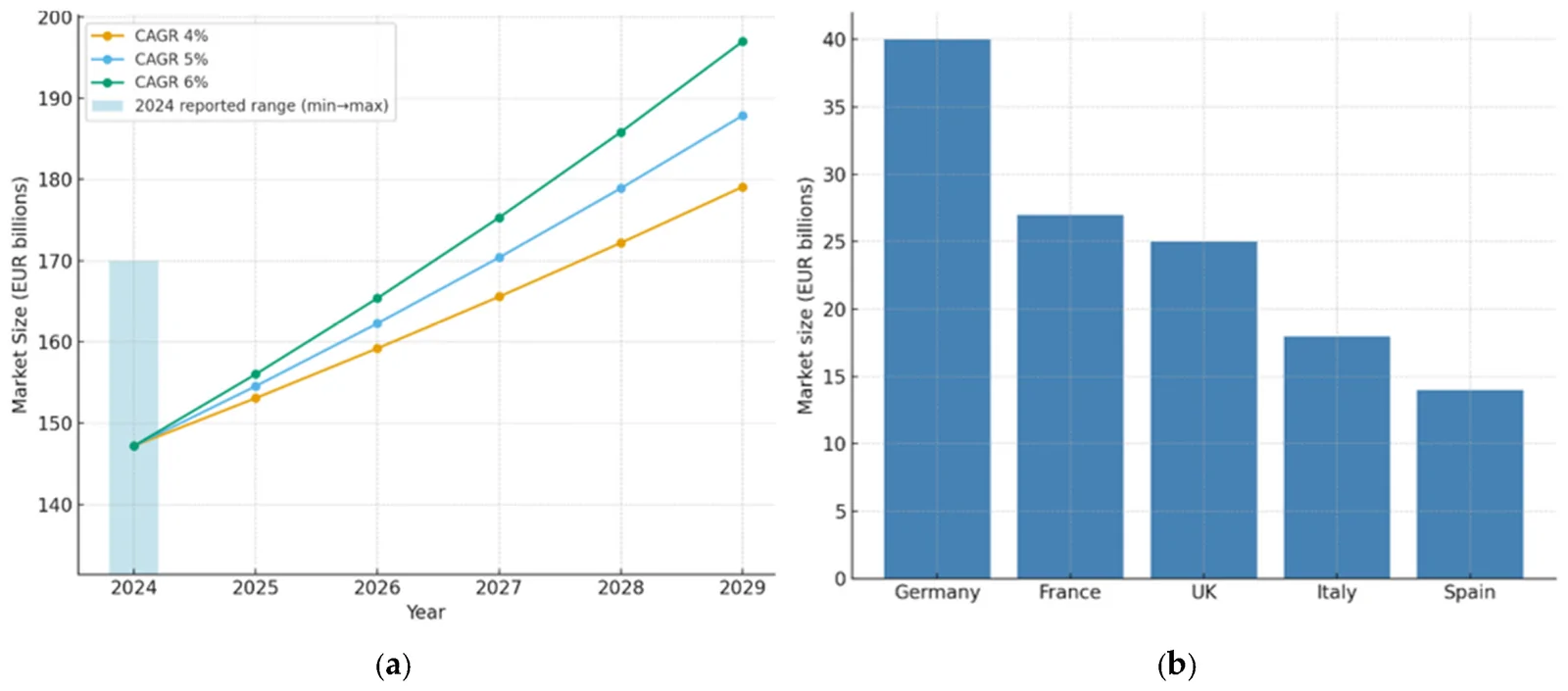
European medical instruments market prospects. (**a**) 2024 reported range of the European medical devices market from [22,23,24] projected to 2029 under three constant-growth scenarios (4%, 5% and 6% CAGR) that bracket the 4–6% range reported by those sources; the scenarios are arithmetic projections of the reported 2024 range and are not forecasts. (**b**) Approximate 2024 national market breakdown from [26,27] for Germany, France, Italy, Spain and, for comparability with the source data, the United Kingdom. Produced in Python 3.11 (Matplotlib 3.8).

The EU medical instruments market, worth €130–170 billion in 2024 and growing steadily, is driven by the top five countries, reflecting differences in health spending and infrastructure. These differences shape where a certified device is purchased and are developed in Section 6 (see Figure 3).

Three properties of this baseline matter for the analysis that follows. The first is the distance between headline growth and segment growth: an aggregate of 4–6% conceals rates ranging from low single digits in mature imaging and consumable categories to sustained double digits in molecular diagnostics, robotics and software-based products, so a statement about “the” European growth rate carries little information unless the segment is specified. The second is the concentration of demand: Germany, France, Italy and Spain, together with the adjacent United Kingdom market, account for the majority of European consumption, which means that national health-expenditure capacity and national procurement practice, not EU-level rules alone, determine where a certified device is actually bought, a point developed in Section 6. The third, and most consequential for this review, is that the baseline measures demand, not supply: it says nothing about how many devices were withdrawn, delayed, or never submitted, which is precisely the quantity the regulatory literature identifies as material. Partial coverage now exists: the Commission’s study supporting the monitoring of the availability of medical devices on the EU market collects repeated notified-body and economic-operator surveys and publishes them through a public dashboard [28]. It is nevertheless survey-based and aggregated and does not provide device-level availability linkable to certification records. This asymmetry between well-measured demand and poorly measured supply recurs throughout the review and is one of the principal evidence gaps identified in Section 12.

Assumptions and transparency:

• 2024 average EUR to USD exchange rate (1.0822) to convert USD-based estimates into EUR, rounding to two decimals for clarity. Exchange-rate choice materially affects the EUR equivalents.
• The three CAGR scenarios are arithmetic illustrations of the 4–6% range reported in [22,23,24], not independent forecasts.
• The scenario projections in Figure 3a are applied to the 2024 range reported by the three independent sources cited in this section [22,23,24]; no additional sources were used for the projection, and no attempt is made to model segment-specific or year-specific growth.

## 4. Regulation: The MDR/IVDR Framework and Its 2025–2026 Recalibration

The regulatory framework for medical devices in the European Union underwent a fundamental overhaul with the adoption of the MDR (Regulation (EU) 2017/745) and the IVDR (Regulation (EU) 2017/746). These Regulations, which replaced the older Directives, aim to strengthen pre-market scrutiny, harmonise safety requirements across Member States, and increase post-market traceability and transparency through a central EUDAMED. The result is a modernised legal architecture that raises the regulatory bar for clinical evidence, vigilance, and manufacturer accountability [29,30].

The MDR and IVDR are directly applicable across the EU, ensuring uniform rules without the need for national transposition. The European Commission, DG SANTE and MDCG coordinate implementation, while national authorities oversee enforcement. Notified Bodies (NBs) handle conformity for higher-risk devices. EUDAMED will serve as the central platform for device registration, unique device identification (UDI), vigilance, and certification [28]. Three sets of changes are most consequential to manufacturers of medical instruments:

• The MDR expands regulatory scope to include more software and non-medical devices, and reclassifies many legacy products into higher-risk categories, requiring stricter conformity [31].
• The MDR demands stronger clinical evidence, ongoing data collection, and systematic evaluation reports, with a focus on real-world and post-market follow-up [15].
• Manufacturers must implement proactive post-market surveillance and safety reporting. UDI and EUDAMED systems will improve traceability and transparency [18].

Fewer Notified Bodies under MDR/IVDR have created capacity bottlenecks, delaying market entry and raising CE certification costs. The Commission, MDCG, and industry groups have responded with pragmatic measures and transitional relaxations to prevent device shortages [29].

To address notified-body shortages and supply risks, the Commission introduced phased timelines, guidance, and deadline extensions for IVDR and legacy devices. MDCG guidance and Commission Q&As are helping firms navigate certification, vigilance, and EUDAMED onboarding, but full MDR/IVDR compliance remains the goal [32]. EUDAMED will centralise device and certificate data, so manufacturers must prepare for UDI labelling and registration, regardless of rollout delays [15]. The shift to MDR/IVDR has real operational and commercial consequences:

• Higher direct compliance costs and resource needs (clinical studies, CERs, technical documentation, notified-body fees).
• Longer certification timelines require rescheduling of launches and portfolio triage.
• Product rationalisation risk: some low-margin legacy devices face withdrawal because recertification is not economically justified.
• Manufacturers, especially small and medium-sized enterprises (SMEs), often report that the new rules require earlier and deeper investment in regulatory affairs, clinical evidence generation, post-market surveillance (PMS) and quality-management resources [33,34].

The framework described above is no longer the framework that will govern the next decade. The evidence accumulated between 2021 and 2025, certification backlogs, withdrawal of low-margin legacy devices, divergent national interpretation and disproportionate pressure on small and medium-sized enterprises was sufficient for the European Parliament to call for revision and for the Commission to open a targeted evaluation, a public consultation and a call for evidence, which together attracted several hundred substantive stakeholder submissions. On 16 December 2025, the Commission published COM (2025) 1023, a proposal amending both Regulations [12]. It is expressly a targeted amendment rather than a rewrite: the fundamental safety and performance requirements are not reopened, and the Commission states that a full impact assessment was not undertaken because the measures are simplification measures for which no viable alternative was identified.

Four elements of the proposal bear directly on the transmission chain examined in this review. First, statutory timelines for the completion of conformity assessment would convert an open-ended process into a bounded one, which is material because duration, and above all the variance of duration, is the channel through which regulation reaches innovation (Section 7 and Section 9). Second, criteria for breakthrough and orphan devices would be introduced, with priority and rolling review, accompanied by guidance published the previous day [35]; this creates, for the first time in the EU, an adaptive pathway of the kind that the comparative literature has repeatedly identified as absent [36]. Third, digitalisation of regulatory procedures and strengthened cooperation between Member States address the inconsistent application that the evaluation identified. Fourth, the proposal sought to clarify the interaction between the device Regulations and the Artificial Intelligence Act by moving the MDR and the IVDR from Section A to Section B of Annex I to that Act, which would have disapplied most of its high-risk requirements to devices. That element did not survive negotiation: under the Digital Omnibus agreed on 7 May 2026 and finally approved by the Council on 29 June 2026, the MDR and the IVDR remain in Section A, AI-enabled devices remain high-risk AI systems subject to dual compliance, and the corresponding obligations are deferred from 2 August 2027 to 2 August 2028 [19]. A parallel instrument, no longer a draft, standardises the quality management systems and procedural requirements of notified bodies [18]; Commission Implementing Regulation (EU) 2026/977 was published in the Official Journal on 5 May 2026 and applies from 25 February 2027. Its Article 2 sets binding maximum timelines for each phase of conformity assessment: 30 days for application review and contract signature, 120 days for quality-management-system audit activities, 90 days for technical documentation assessment and 20 days for the certification decision and issuance, including EUDAMED registration, and Article 3 caps the number of permitted interruptions per phase. Statutory timelines are therefore already law, and do not depend on adoption of the December 2025 proposal. Article 4 requires every notified body to operate a system that monitors the duration and cost of its conformity assessment activities and, from 1 January 2028, to publish annually the proportion of activities completed within the maximum timelines, the median duration from application to certification, and the median total cost. This is directly relevant to the present review: it will produce, for the first time, an independently reported measure of the quantity that Section 7 can currently document only through industry self-report. The Regulation addresses variability in assessment practice rather than notified-body capacity as such.

Three qualifications are essential. The proposal is not law: it must pass the European Parliament and the Council, and the classification rules and AI Act interface are the provisions most likely to be renegotiated [12]. It does not itself create notified-body capacity, so statutory timelines will bind only if throughput permits, and industry bodies have framed the proposal as a first step rather than a resolution [13]. And it contains no provision compensating the patent life consumed by conformity assessment, which is the mechanism through which regulatory duration is currently estimated to affect development incentives [16]. Meanwhile, the transitional architecture of Regulation (EU) 2023/607 remains in force: legacy Class III and implantable Class IIb devices must be certified by 31 December 2027 and non-implantable Class IIb, Class IIa and up-classified Class I devices by 31 December 2028, conditional on an MDR application having been lodged by 26 May 2024 and a notified-body contract signed by 26 September 2024 [37]. The recertification wave therefore peaks during the same period in which the revision, if adopted, would take effect.

The transparency architecture has moved in parallel. EUDAMED’s actor, device and certificate modules carry mandatory obligations from 28 May 2026, requiring registration of economic operators under a single registration number, registration of device models with their unique device identifiers before placing on the market, and upload of notified-body certificates [15]. For manufacturers, this is an additional data-management obligation. For this review, it is more significant as infrastructure: EUDAMED is the first instrument capable of producing a public, device-level record of what is certified and available in the Union, and therefore the first instrument capable of measuring the healthcare-system consequences that the Regulations are intended to secure. That it became mandatory only in 2026, nine years after adoption of the Regulations, is itself part of the explanation for why the terminal link in the framework chain remains so poorly evidenced.

## 5. Evidence Intensity and Segment Heterogeneity

In 2024, the largest product and therapy areas are consistently reported as IVD, diagnostic imaging, and cardiology or implantable devices, including orthopaedics. Some analyses count reagents and software together with instruments in IVD, while others present separate figures for these components. Despite these differences, the overall consensus of MedTech Europe [38], Grand View Research [25], Data Bridge Market Research [39], Market Data Forecast [40], and other providers are that mature categories such as imaging and surgical equipment typically grow in the mid-single digits, while robotics, molecular IVD, wearables and other innovation-intensive segments can sustain double-digit growth trajectories. MedTech Europe identifies IVD as the single largest sector within European MedTech, followed by cardiology-related implantable and diagnostic imaging [41]. Overlaps between categories are inevitable, as with IVD reagents compared to diagnostic instruments, or disposables compared to single-use devices.

Because this review treats evidence intensity as an explanatory variable, it must define the term before using it. Table 1 sets out the classification applied throughout. It is a rule-based mapping, not a score: each segment is assigned the risk class and conformity-assessment route that predominates within it under Annex VIII of the MDR and of the IVDR, together with the resulting level of notified-body involvement and clinical evidence requirement. Segments are heterogeneous internally, and the table records the predominant case only. That limitation is material to the analysis below.

**Figure 4:**
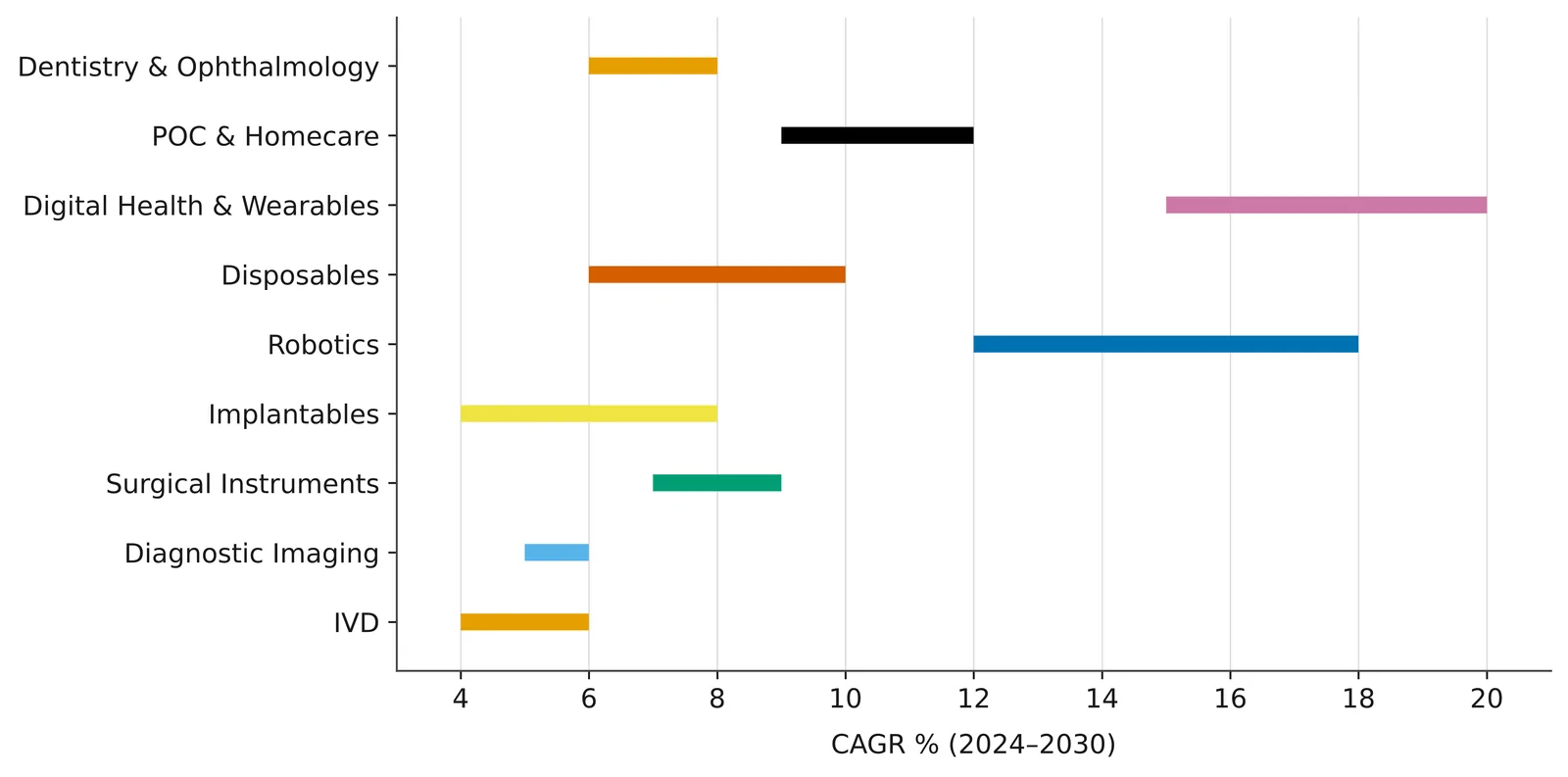
EU medical instruments market: comparative CAGR ranges by segment, 2024–2030. Ranges compiled by the authors from [25,26,27,28,29,30,31,32,33,34,35,36,37,38,39,40,42,43]; where providers disagree, the full reported span is shown. Produced in Python 3.11 (Matplotlib 3.8).

In vitro diagnostics (IVD) are Europe’s largest MedTech segment, valued at $23–32 billion in 2024 [42,43]. Growth is steady at 4–6% CAGR, as shown in Figure 4, with molecular diagnostics and point-of-care (POC) tests rising fastest due to decentralised care, infectious disease preparedness, and chronic disease burden [44]. The IVDR raises compliance costs and strains notified-body capacity, but firms meeting these standards can lead to molecular POC, digital IVD, and AI-enabled diagnostics [41].

**Figure 5:**
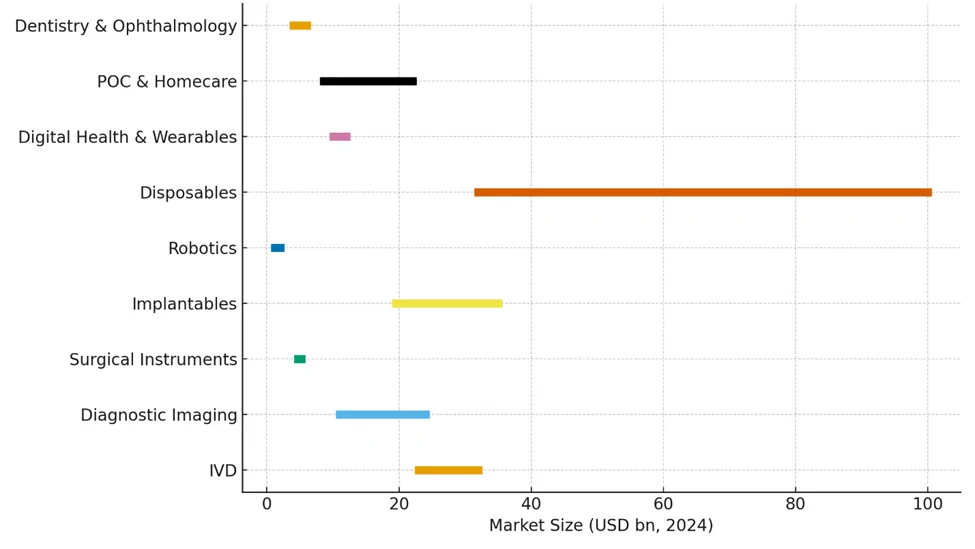
EU medical instruments market: comparative segment value estimates, 2024. Values compiled by the authors from [25,39,42,43]; segment scopes differ between providers, in the treatment of IVD reagents relative to instruments and of single-use devices relative to disposables, so the bars are not mutually exclusive and should not be summed. Produced in Python 3.11 (Matplotlib 3.8).

Diagnostic imaging is a core segment of Europe’s MedTech market, valued at $11–24 billion in 2024 (Figure 5), depending on the scope [25,39]. Imaging grows at a 5–6% CAGR, with faster gains in advanced modalities such as hybrid PET/MR, spectral CT, and AI-powered software. Key drivers include rising demand in oncology, AI adoption, and the need to replace outdated equipment. Siemens Healthineers, Philips, and GE HealthCare lead the high-end imaging market [45].

Implantable devices represent another cornerstone of European MedTech. This category encompasses cardiology and vascular implants, such as pacemakers and stents, as well as orthopaedic implants, including hip and knee replacements, and dental implants. Orthopaedics alone accounts for an estimated 19–20 billion USD in Europe in 2024 [39]. Ageing demographics support growth in orthopaedics and cardiology, expanded clinical indications and material innovation, including 3D printing. Challenges include heightened regulatory scrutiny, health technology assessment requirements and supply chain resilience issues [42].

Surgical robotics in Europe is still a small market but is growing rapidly, with double-digit annual growth driven by better patient outcomes, falling costs, and SaaS or consumable-based revenue models [46,47]. Estimates for disposables and single-use devices span USD 30–100 billion (approximately EUR 28–92 billion) for 2023–2024, with reported growth of 6–10%. The upper bound of that span approaches two-thirds of the entire European market quoted in Section 3 and is irreconcilable with identifying in vitro diagnostics as the largest single segment; it reflects a definition of “disposables” that extends well beyond devices regulated under the MDR. The range is reported here only to document that disagreement, and is not used in any comparison [25]. However, sustainability concerns and circular-economy demands are rising challenges for both segments.

Figure 4 and Figure 5, read against the classification in Table 1, do not support the proposition that growth is ordered by evidence intensity. The association at the segment level runs the other way. The two segments with the heaviest evidence burden in Table 1, implantable devices and in vitro diagnostics, report the lowest growth ranges in Figure 4 (approximately 4–8% and 4–6% respectively), while the fastest-growing segments, digital health and wearables at approximately 15–20%, sit predominantly in the lower conformity-assessment classes and in some cases outside the Regulations altogether. Surgical robotics is the one high-evidence segment with double-digit growth, and it does so from a small base. Therefore, based on the evidence assembled here, the descriptive claim that the most evidence-intensive segments grow fastest is not sustainable, and we withdraw it.

The inverse association is itself informative, but it must be interpreted with care, and three qualifications limit how far it can be taken. First, the data are at segment level while the regulatory burden attaches to individual devices; sub-segments such as molecular in vitro diagnostics or implantable cardiology may well behave differently from the segment aggregates shown, but the commercial sources do not report growth at that resolution, so the proposition cannot be tested with the data available. Second, growth rates and evidence intensity are not independent of base size: the low-burden, high-growth segments are generally the smaller and newer ones, and growth expressed as a percentage rewards a small base. Third, the direction of causation is not identified. A negative association between evidence intensity and growth is consistent with regulation suppressing growth in the most regulated segments, but equally with maturity, replacement-cycle economics and demographic saturation in those same segments. No study in the retained corpus separates these explanations. The relationship is therefore graded in Section 11 as an observed association of uncertain sign at device level, not as a demonstrated mechanism.

The classification does support a narrower, better-evidenced claim about participation rather than growth. Expected return in any segment must be discounted by a certification hazard measurable in months of delay and in the probability of an iterated or negative assessment, and that discount rises with the evidence intensity recorded in Table 1. It falls disproportionately on firms without in-house clinical and regulatory capacity, which is to say on the small and medium-sized enterprises that constitute the large majority of the European industry by number of companies [48]. The consequence is compositional: in the most evidence-intensive segments, the set of firms able to participate narrows, irrespective of whether the segment itself grows quickly or slowly. For healthcare providers and patients, the resulting exposure is to notified-body throughput in exactly those segments where devices are least substitutable. What remains unresolved is magnitude: no public dataset links device-level certification duration to segment-level revenue in the Union, and the quantitative statements in this section are therefore directional rather than elastic.

Europe’s medical instruments market is both concentrated and fragmented. IVD and imaging are dominated by global leaders like Roche, Siemens, Philips, and Abbott [47,49], while surgical instruments, disposables, and digital health remain split between multinationals and SMEs, with ongoing consolidation and competition from start-ups [25]. Mature segments face margin pressure under limited regulatory risk, whereas molecular IVD, robotics and software as a medical device combine higher expected returns with the evidence requirements recorded in Table 1.

## 6. Demand-Side Drivers and Their Interaction with the Regulatory Chain

**Figure 6:**
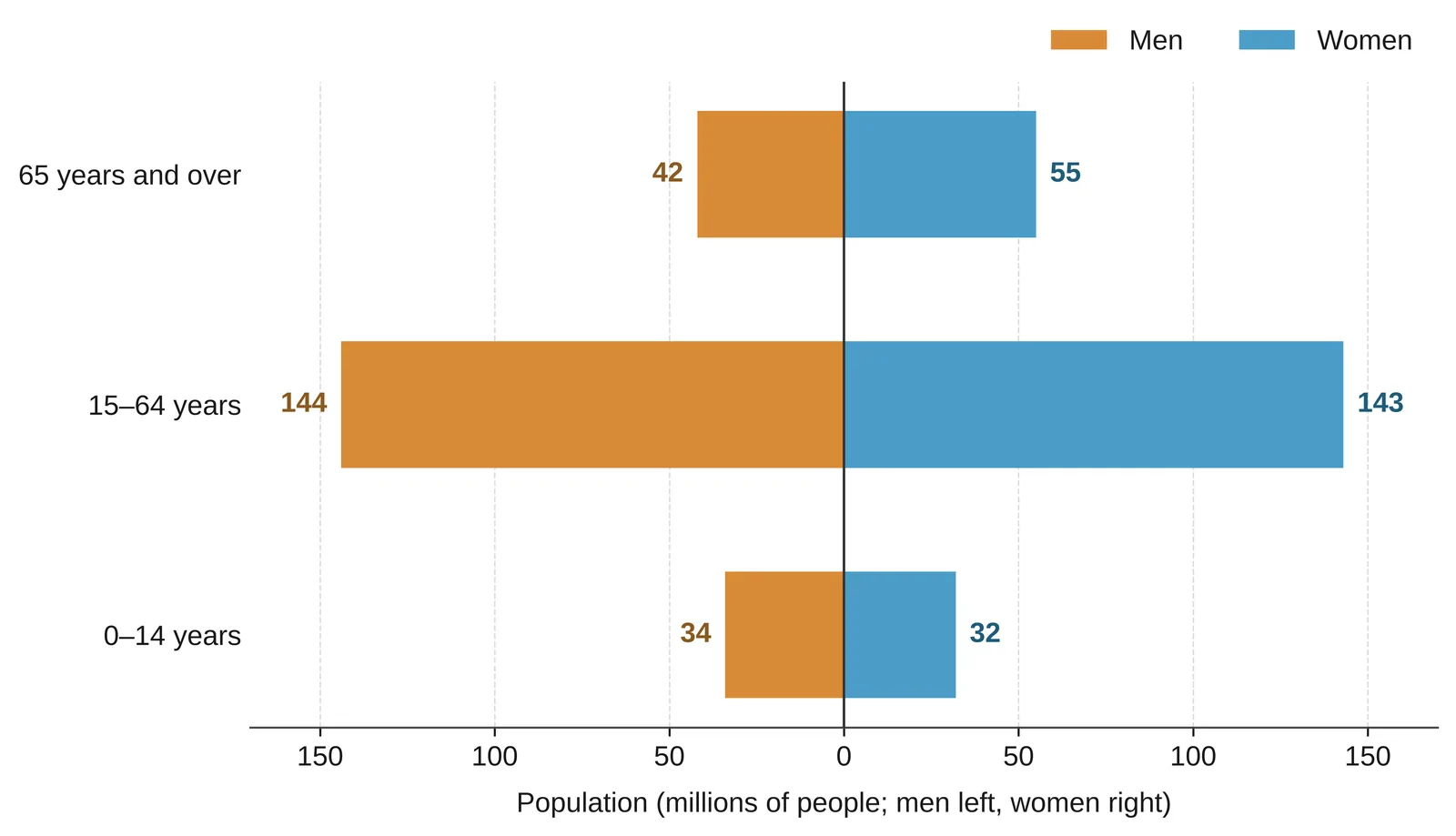
EU population structure by broad age group, 1 January 2024. Data from Eurostat [50]; the horizontal axis shows population in millions, and the two series are men and women within each age group, not a time trend.

A small group of powerful, interacting drivers shapes the European medical instruments market, influencing both current demand and future opportunities. The primary structural driver is demographic change: Europe is ageing rapidly, and the proportion of older adults is large and continues to grow, increasing demand for diagnostics, chronic-care devices, implants, and long-term monitoring technologies (see Figure 6). Eurostat reports that on 1 January 2024, the EU population was about 449.3 million, with more than one-fifth (21.6%) aged 65 years or over; these demographic trends are ongoing and will significantly boost the volume of procedures and long-term device usage in the coming decades.

Europe’s high burden of non-communicable diseases—cardiovascular, cancer, diabetes, and respiratory—drives ongoing demand for diagnostic and therapeutic devices. WHO data show NCDs cause most deaths and illnesses in Europe, making them a key reason for strong MedTech demand in imaging, IVD, cardiology, and orthopaedics [51].

**Figure 7:**
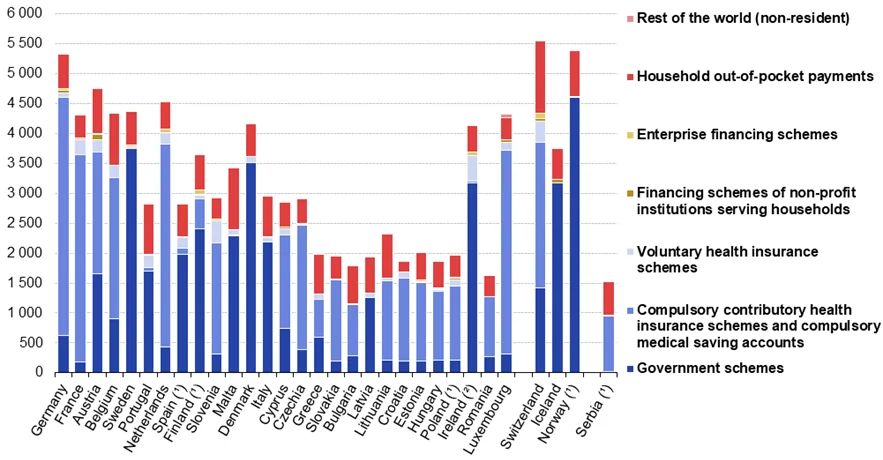
Current healthcare expenditure per inhabitant, analysed by source of financing, 2022, in PPS per inhabitant (adopted from Eurostat [47]). Note: ranked on current healthcare expenditure as a percentage of GDP. ^1^ Provisional; ^2^ Enterprise financing schemes also include financing of non-profit institutions serving households.

The second major economic driver is public and private health spending. Aggregate healthcare expenditure in the EU is substantial; Eurostat reports current healthcare expenditure of approximately €1648 billion in 2022, equivalent to about 10.4% of GDP, and national spending patterns (for example, Germany and France) are among the highest in Europe (see Figure 7), and these budget levels determine the fiscal space for new devices, capital renewal and service contracts [52].

The OECD reports that, despite short-term swings, health spending across OECD countries has continued to rise in both absolute terms and as a share of GDP, and projects that ageing populations will sustain demand for new technologies, capital equipment and recurring revenue items [53].

**Figure 8:**
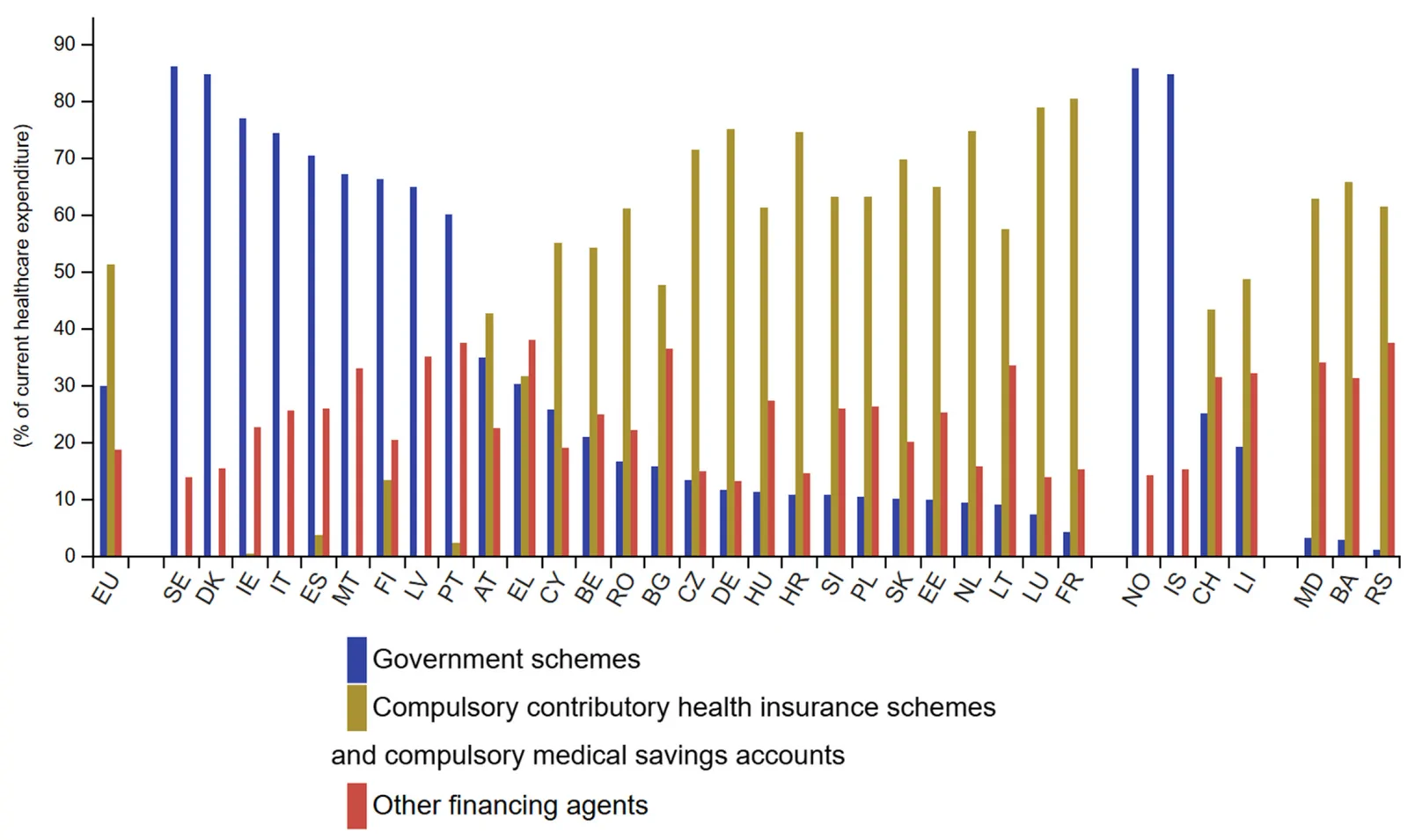
Main sources of financing of current healthcare expenditure, 2022 (adopted from Eurostat [52]).

Digitalisation and AI are rapidly transforming MedTech. Software-driven features, cloud diagnostics, and data connectivity are shifting revenue to subscriptions, outcome-based contracts, and data monetisation. McKinsey reports that digital tools, including generative AI, are reshaping product strategies and business models [54]. For device vendors, this creates both new revenue opportunities and a greater need for expertise in data, cybersecurity, and regulatory compliance for software-as-a-medical-device. The major sources of financing for current healthcare expenditure are presented in Figure 8.

Regulatory change is reshaping MedTech in Europe. The MDR and IVDR raise standards for evidence and traceability, protecting patients but also increasing compliance costs and entry barriers, especially for smaller suppliers [55]. Tighter rules boost demand for high-quality devices and compliance services, making regulation both a cost and a source of new market value. EUDAMED and MDCG guidance further define these evolving requirements.

Decentralisation is transforming care in Europe, with health systems moving diagnosis and chronic disease management into outpatient, community, and home settings. The rise in point-of-care diagnostics, remote monitoring, and wearables, accelerated by COVID-19 and supportive reimbursement, drives demand for compact, user-friendly devices and integrated digital platforms [54].

Procurement, reimbursement, and HTA rules are shaping device adoption and pricing in Europe. Value-based procurement is gaining ground, with HTA bodies demanding strong clinical and economic evidence, forcing manufacturers to invest in real-world data to secure market access [53]. Centralised and regional procurement creates a complex landscape that requires tailored strategies. The pandemic exposed supply chain vulnerabilities, prompting the EU to prioritise strategic autonomy and resilience. Manufacturers now diversify suppliers and nearshore production to win tenders for high-value devices. Sustainability is also gaining importance: The European Green Deal and new policies are pushing for reduced waste and greater circularity. Devices with lower environmental footprints are favoured, and manufacturers must document emissions and compliance. MedTech Europe is working with policymakers to guide sustainability standards for medical devices.

Scale, evidence, and technology now determine success in Europe’s device market. Demographic trends drive steady demand for diagnostics, implants, and chronic-care devices [50], while procurement, digitalisation, and regulation shape growth and competitiveness. Winning manufacturers pair clinical and economic evidence with digital strength, supply-chain resilience, and sustainability, aligning with procurement and HTA requirements. Those who master these dimensions will lead Europe’s device market in the years ahead.

## 7. Evidence Generation, Certification Duration and the Compliance Burden

Europe’s medical device market faces complex, structural challenges: regulatory changes, rising compliance costs, supply chain fragility, tougher procurement, and new demands in cybersecurity, workforce, and sustainability. These pressures affect every aspect of market and product strategy. The MDR and IVDR present the most urgent hurdle, requiring stronger evidence, ongoing surveillance, and traceability, raising the bar for CE marking and increasing workload and costs [56]. Many manufacturers, especially SMEs, now face long certification delays, higher fees, and repeated technical hurdles. These operational burdens have sparked EU-level debate on how to address assessment bottlenecks and protect supply continuity.

A shortage of NBs for MDR/IVDR conformity assessment has created long queues, delayed certificates, and forced some manufacturers to withdraw lower-margin products, raising time-to-market, compliance costs, and patient safety concerns [57]. This bottleneck benefits larger firms and threatens niche devices. At the same time, supply-chain fragility and geopolitical shocks have driven up input costs and lead times for medical instruments. The 2021–2022 semiconductor shortage exposed vulnerabilities, prompting policymakers to focus on resilience and strategic sourcing [58]. Now, manufacturers must weigh higher costs against operational risk when deciding whether to near-shore or dual-source production.

Tight budgets and strict procurement rules make it hard for MedTech firms to pass on rising regulatory and manufacturing costs. EU and national HTA processes now demand strong clinical and economic evidence, raising market access barriers and making clinical trials, real-world evidence, and health-economic modelling essential for reimbursement [59]. These requirements especially disadvantage smaller firms with limited resources, deepening the gap between large and small competitors.

Digitalisation and connected devices boost MedTech’s value but bring new regulatory, cybersecurity, and data privacy challenges. Regulators now require secure-by-design products and strong vulnerability management, raising compliance costs and demanding greater IT and data expertise from manufacturers [60]. Meanwhile, staff shortages in hospitals and MedTech firms mean solutions that save clinician time or automate tasks are in high demand. Firms must invest in training and support to win contracts, but these costs create barriers for newcomers [61].

Sustainability and circular economy demands are reshaping MedTech. The European Green Deal and new procurement rules require proof of low emissions, recyclability, and waste reduction [62]. Single-use devices face intense scrutiny, and firms must document their environmental impact and offer reuse or recycling options. Those lacking credible sustainability risk exclusion from tenders and from cost-driven contracts.

Europe’s MedTech market is split between powerful global incumbents and fragmented segments of small specialists. While niche innovators offer acquisition potential, they face steep regulatory and procurement barriers to scale. Ongoing consolidation may concentrate market power and raise costs for health systems [63]. Meanwhile, parallel imports and counterfeits threaten supply integrity and complicate pricing, adding further challenges for manufacturers and regulators. Legal risk is rising as MDR and IVDR demand stronger clinical evidence and transparency, increasing liability if safety issues emerge after market entry [64]. Manufacturers must invest in quality systems, post-market surveillance, and rapid corrective actions, especially smaller firms facing higher insurance and capital costs. The most directly measured quantity in the transmission chain is the conformity assessment duration itself. The figures below are taken from the MedTech Europe IVDR and MDR Survey 2024 public report [64], a voluntary industry survey of manufacturers with certificates or applications in progress. Phase figures are medians as reported by the survey; totals are averages across subgroups and therefore not the sum of the phase medians, which cannot be added. Both are reproduced as published, with the resulting inconsistencies identified after the lists.

For In Vitro Diagnostic (IVD) conformity assessments:

• The median Notified Body Pre-Review Phase (from application submission to review start) is about 4.6 months for large companies and 4.5 months for SMEs in TDA (Technical Documentation Assessment) contexts; elsewhere, MedTech reports a median pre-review of 5.4 months (large) vs. 8.8 months (SMEs) for quality management system (QMS), depending on the dataset and sample. The specifics vary by QMS vs. TDA and by IVD vs. MD.
• The median Review time (application review start to positive recommendation) is about 8–9 months (8.3 months for large companies and 9.3 months for SMEs in the TDA sample).
• The median Certificate issuance time (from positive recommendation to certificate issuance) is ~4.9 months for large companies and 2.8–4.0 months for SMEs, depending on the dataset.
• Total time for IVD certification (QMS or TDA) is reported by the survey as an average, clustering around 17–19 months, with individual subgroup totals of 18.5 and 17.3 months depending on sample and averaging method [64].

For Medical Devices conformity assessments under MDR:

• The median pre-review phase for QMS is approximately 6 months for large companies and 4 months for SMEs in one sample. For TDA, the median pre-review phase is 6 months (large) and 3 months (SMEs).
• The median Review time for MD TDA is longer than IVD: around 13–14 months (e.g., 13.7 months for large companies and 14.3 months for SMEs in one dataset), while QMS review medians are around 10–10.5 months for larger companies.
• The median certificate issuance phase is typically around 3.8–4.6 months, depending on the cohort.
• Total time for MD conformity assessment (QMS and TDA combined) is reported by the survey as an average of 19–22 months, with subgroup totals of 19.5 to 21.8 months [64].

Four features of these figures matter more than their headline values, and the first is a caveat rather than a finding. The phase medians and the reported totals are not arithmetically consistent. Summing the MDR technical-documentation phases for large companies gives about 24 months (6.0 + 13.7 + around 4.0), which is above the 19–22 month total the survey reports, whereas the corresponding IVDR sum of about 17.8 months (4.6 + 8.3 + 4.9) falls within the reported 17–19 months. Part of the discrepancy is expected because medians are not additive and the totals average partly different subgroups, but the source does not explain the direction or size of the MDR gap. The 19–22 month figure used in this review and in its abstract is therefore the survey’s own reported total and should be read as a lower bound for the technical documentation route for large companies.

Second, the phases that consume the most time are not the technical assessment itself: pre-review and certificate issuance together account for a substantial share of the total, and both are administrative rather than scientific. That is why both the December 2025 proposal [12] and, decisively, Implementing Regulation (EU) 2026/977 [18] target procedure and deadlines rather than evidence requirements. Third, the dispersion between large enterprises and SMEs is not uniform across phases: SMEs fare comparably or better in some pre-review and certificate-issuance samples but worse in review, which is consistent with file completeness and clinical-evidence depth, rather than firm size as such, being the operative variable.

Fourth, this review has argued elsewhere that certification duration variance matters more to investment decisions than its mean. That proposition is a hypothesis, not a measurement, and it is stated as such here: the survey reports medians and averages, but not dispersion, and no public dataset currently reports the distribution of certification durations. The hypothesis is nonetheless the one on which the regulator has acted, since both the maximum timelines of Article 2 and the interruption caps of Article 3 of Implementing Regulation (EU) 2026/977 address predictability rather than average speed [18]. It will become testable: from 1 January 2028 every notified body must publish the proportion of activities completed within the maximum timelines and the median duration from application to certification, which provides the independent, non-self-reported series that the present evidence base lacks and that is the principal qualification on R1 in Section 11.

A second evidence filter is now being added downstream. Under the Health Technology Assessment Regulation [65] joint clinical assessments were applied first to oncology medicines and advanced therapies from January 2025, with the first report adopted on 30 April 2026 [65]. High-risk devices entered scope in 2026, restricted to Class IIb and Class III devices and Class D in vitro diagnostics that have passed expert-panel consultation during CE certification, with approximately five assessments planned in the first year alongside two to five joint scientific consultations [14]. The first selection was made on 6 October 2026, when the Commission designated two cardiovascular devices, both mitral valve replacement systems, for the first device joint clinical assessments, following a recommendation of the Coordination Group of 22 June 2026; further selections are expected quarterly [20]. The scale is deliberately small, and both first-selected devices fall in the highest evidence-intensity band of Table 1, but the structural significance is not small: for the first time the clinical narrative supporting a high-risk device will be assessed once at Union level, even though reimbursement remains national. Interview evidence from national HTA bodies indicates broad willingness to use joint assessment output but persistent uncertainty about timing, selection and contextualisation within national reimbursement processes [52]. For manufacturers, the practical effect is that evidence planning must now satisfy two assessors with different questions: a notified body asking whether the device is safe and performs as claimed, and an HTA body asking whether it is clinically better than what is already reimbursed, and must do so from the same clinical programme, designed years earlier.

Investor appetite and access to capital shape how MedTech firms respond to regulatory risk, reimbursement uncertainty, and supply-chain challenges, factors that drive valuations and funding availability. Well-capitalised firms can invest in clinical programmes, manufacturing, cybersecurity, and sustainability, giving them a competitive edge in Europe. Smaller companies often face high compliance costs and slow market entry, making acquisition by larger players the main route to scale [64]. The EU market’s complexity, from regulatory reform to trade uncertainty, demands robust clinical evidence, resilient manufacturing, and integrated digital and sustainability strategies for commercial success.

## 8. Market Access and Competitive Selection: Capability as the Binding Constraint

Europe’s medical instruments market is dominated by a few global giants and a long tail of specialised SMEs. High-value segments like IVD, imaging, and implants are highly concentrated, while niche devices remain fragmented [63]. Large players, such as Roche, Abbott, Siemens Healthineers, bioMérieux, and Danaher, lead IVD with integrated platforms and high switching costs, securing recurring revenues and durable advantage [66]. In imaging, Siemens Healthineers, GE HealthCare, and Philips control the market, leveraging scale and service networks for a strong edge. Although software and AI are reshaping pricing, incumbents’ installed bases and long-term service contracts remain powerful barriers to entry [67].

The European orthopaedic device market is dominated by a few major players, Stryker, DePuy Synthes, Zimmer Biomet, and Smith & Nephew, who control most joint-reconstruction and spine segments and bundle implants with capital equipment, instruments, and digital solutions. Stryker’s 2024 strategy and market data highlight a focus on acquisitions to fill product gaps, while the top suppliers collectively capture most reconstruction revenues in Europe [68]. This concentration raises barriers for newcomers, who need compelling clinical evidence or acquisition to compete. In surgical robotics, Intuitive Surgical’s da Vinci system maintains global dominance with a large installed base and strong recurring revenues; other giants and new entrants target speciality and cost-sensitive niches [69]. Entrenched leaders use recurring revenue and scale to defend margins, while challengers must offer clear differentiation to gain traction.

Despite the prominence of large firms, SMEs account for about 95% of European medical technology companies by number, but a much smaller share of turnover [48]; the distinction matters because compliance costs scale with the number of product families rather than revenue. However, they face significant disadvantages in terms of compliance costs and access to clinical evidence, which fuel ongoing consolidation and partnerships. Mergers and acquisitions remain key to competitive strategy: 2024 saw major deals and targeted tuck-ins, especially in orthopaedics, cardiology, and digital health, as firms seek platform scale and expanded service offerings. Industry reporting records 2024 as a year of high-value transactions concentrated in orthopaedics, cardiology and digital health [70].

Private equity and strategic investors are fuelling MedTech consolidation in Europe, attracted by steady service revenues and the chance to roll up fragmented niches. This trend boosts competition for assets and offers exit options for SMEs but also drives up valuations and accelerates sector consolidation [71]. In digital health, software as a medical device (SaMD), and AI, software markets scale rapidly through platform effects. Hospitals now favour integrated device-software solutions, benefiting firms with proven, interoperable platforms. This demand drives acquisitions and partnerships, as large device makers acquire digital capabilities rather than build them from scratch. As a result, stand-alone software vendors face consolidation, with industry reports highlighting a surge in software deals and partnerships [70].

Aftermarket services and consumables generate high-margin, recurring revenue, giving incumbents a powerful edge and incentivising long-term customer contracts. This “platform-plus” model raises switching costs, making it tough for newcomers to compete without partnerships or disruptive pricing. Major players like Siemens Healthineers, GE HealthCare, and Philips rely on service revenue as a key strategic moat. Regulatory changes, especially MDR, IVDR, and the EU HTA, raise entry costs and reward firms that invest in evidence and compliance. Larger companies can absorb these costs, while smaller firms often turn to partnering or portfolio rationalisation [72]. These trends reinforce scale advantages and drive consolidation, as highlighted by industry surveys and policy trackers.

Geopolitical shifts and supply chain disruptions are prompting companies and governments to invest in local manufacturing of critical medical equipment, thereby boosting supply resilience and competitive positioning [73]. Firms that can prove regional sourcing or reliability are favoured in procurement. Regional champions thrive by leveraging local networks and clinical expertise, while international players succeed by partnering with domestic leaders. Success in Europe requires tailored, country-by-country strategies rather than a one-size-fits-all approach, given the diversity of national procurement and clinical pathways [23].

The competitive pattern described above is the market-structure signature of the evidence requirement rather than of technology or capital alone. Where conformity assessment and health technology assessment both demand clinical and health-economic evidence, the capability to generate that evidence becomes a barrier to entry in its own right, independent of product quality: a device that would satisfy the essential requirements may still fail to reach patients because its manufacturer cannot finance the evidence needed to prove it. This is the mechanism behind three otherwise separate observations: the persistence of incumbent advantage in IVD and imaging, the dependence of small specialists on acquisition rather than organic scale, and the growth of regulatory and clinical-affairs services as a market in their own right. Regulatory tightening and industry consolidation have moved together over the same period. This review does not claim that the first caused the second. Consolidation in medical technology predates the MDR and is driven by platform economics and recurring service revenue as well as by compliance cost; no study in the retained corpus separates the regulatory contribution from the rest, and no counterfactual is available. The relationship is reported here as a co-occurrence consistent with the evidence-capability mechanism, and it is graded accordingly in Section 11.

## 9. Innovation: Measurable Consequences of Regulatory Change

Innovation in European medical devices is accelerating, driven by digital technology, decentralised diagnostics, and new value models for software and data. Digital and AI solutions [74], robotics [75], point-of-care diagnostics [76], additive manufacturing [77], and software-first platforms [78] are reshaping product strategies and procurement. The digital shift is especially powerful: AI and advanced analytics are moving value from hardware sales to ongoing software and workflow services, as hospitals increasingly pay for automated interpretation and device optimisation.

MedTech companies are shifting to software-driven products, data services, and subscription models, with digital capabilities like imaging AI and cloud diagnostics expected to drive future revenue growth [54]. Generative AI is streamlining clinical workflows and boosting efficiency across Europe. Surgical robotics and automation are also advancing fast: while still a small part of surgical equipment spending, their use is growing quickly, fuelled by integrated platforms and national initiatives to modernise surgery. As robotic systems become more common, competition intensifies. First movers build strong market positions, while new, lower-cost entrants target broader adoption beyond elite hospitals [79].

Point-of-care diagnostics and molecular testing are transforming European healthcare, enabling faster, near-patient results and immediate clinical decisions. The market for molecular POC is expanding, especially in infectious disease, oncology, and genetic screening, though IVDRs raise conformity costs even as they build trust in high-evidence devices [80]. The push for decentralised, robust diagnostics is strong. Meanwhile, additive manufacturing is moving from research to mainstream clinical use. 3D printing now enables complex, patient-specific implants and surgical guides, while advances in materials, process control, and regulatory clarity reduce adoption barriers. Machine learning and closed-loop systems are making printed implants more reliable and biocompatible, and new printing methods are expanding possibilities [81,82]. With rapid growth and ongoing research, personalised implants and on-demand manufacturing are poised to reshape speciality device markets in Europe.

Software-first business models, data-driven platforms, and service bundling are transforming competition in the medical device industry. Companies now win by offering data aggregation, outcome tracking, and value-based contracts, not just mechanical performance. Firms that capture patient-level data and prove economic value gain an edge, while partnerships between hardware and leading digital players accelerate innovation [83]. Interoperability, digital twins, and simulation further differentiate offerings, enabling remote monitoring, predictive maintenance, and advanced clinical planning. To succeed, companies must prioritise open standards, validated interoperability, and strong data governance.

**Figure 9:**
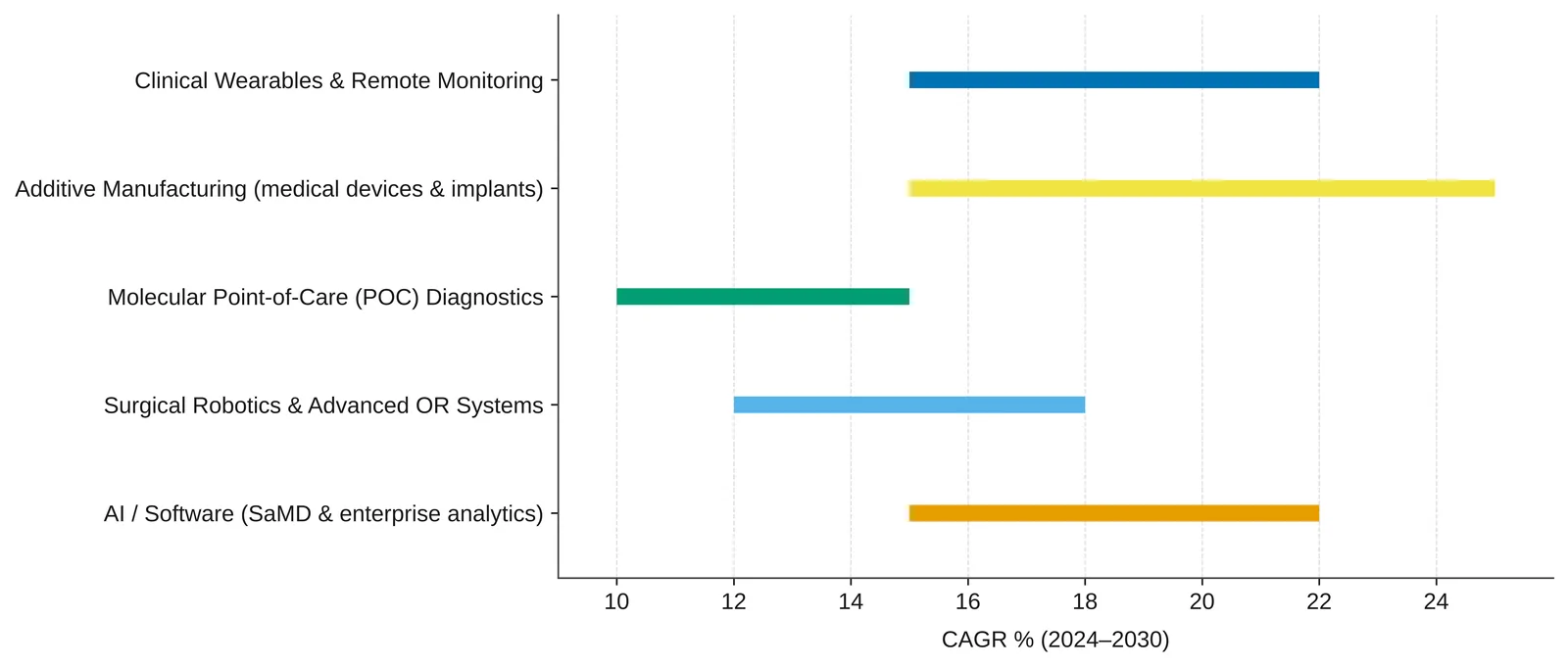
CAGR ranges for innovation topics 2024–2030.

Cybersecurity and data privacy are now essential for medical device innovation. As connected devices and AI increase risks, regulators and buyers demand proof of robust security and lifecycle management. Firms lacking strong cybersecurity face delays, higher costs, and reputational harm. Evidence standards are also rising; authorities now expect real-world outcomes and health economic data, not just technical specs. Companies must invest in evidence generation and post-market data collection to win contracts and achieve reimbursement. IVDR and MDR reforms make ongoing surveillance and traceability a must for market success. The IVDR and MDR reforms accentuate this reality by explicitly tightening post-market surveillance and traceability obligations. CAGR ranges for innovation topics projected for 2024–2030 are presented in Figure 9.

Stricter MDR and IVDR standards raise costs but also reward high-quality, evidence-backed innovations. Products with traceability, real-world data, and clear patient benefits stand out. Early regulatory planning and digital surveillance help firms secure and sustain market access. Buyers now demand solutions that quickly improve efficiency or outcomes, not just incremental advances. To win contracts, vendors must prove device performance, interoperability, cybersecurity, and measurable results. Success requires tightly aligned product, evidence, and commercial strategies.

**Figure 10:**
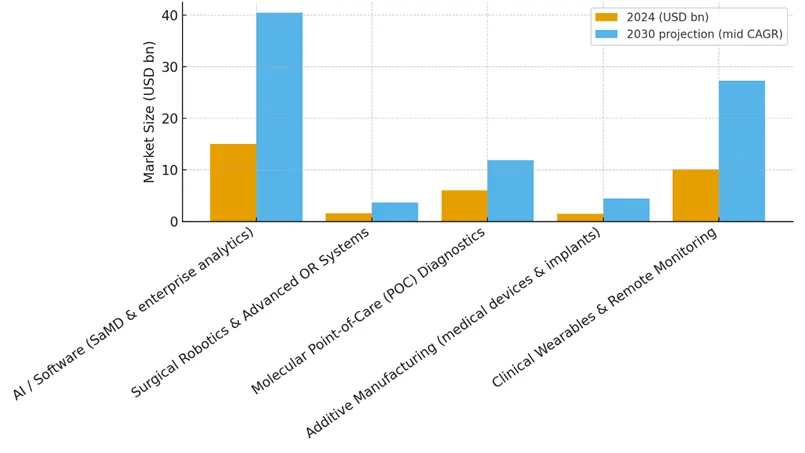
Innovation topics: 2024 baseline vs. projection (midpoint CAGR).

The comparison between the 2024 market size baseline and the projected midpoint CAGR for 2030 demonstrated the economic potential of different innovation topics (see Figure 10).

Until recently, the proposition that MDR-related delay reduces innovation rested largely on self-report. Two 2026 contributions change the evidentiary status of that claim. The first is an explicit economic model of development incentives under the EU and US frameworks, formulated as a continuous-time optimal-control problem in two markets with a spillover channel representing the cross-jurisdictional reuse of clinical evidence and quality systems [16]. Its central observation is structural rather than rhetorical: patent terms run from filing, whereas conformity assessment consumes time after filing, so any lengthening of the assessment path shortens effective patent life. Unlike medicinal products, EU medical devices have no statutory mechanism, no analogue of the supplementary protection certificate to restore that time. The model quantifies the resulting incentive loss and estimates that introducing a three-year device supplementary protection certificate limited to Class III devices would raise EU-directed development effort by 13.46% (95% confidence interval 12.8–14.1%) in a single-market EU calibration. The estimate is model-based, and its magnitude depends on calibration, but its direction is identified by an explicit mechanism, which goes beyond what the descriptive literature has previously offered.

The second is a survey of European device manufacturers spanning micro to large enterprises, conducted after the amending regulation and the successive transitional extensions [17]. It reports that manufacturers increasingly seek initial approval and launch outside the EU, and concludes that, despite the mitigations adopted to reduce burden and protect availability, the EU is no longer the market of choice for the first introduction of new products. This is a statement about the location of innovation rather than about its volume, and it is precisely the outcome the model predicts: when regulatory delay is not compensated, development effort reallocates towards the jurisdiction in which the effective protected period is longer. An older quasi-experimental study of United States Food and Drug Administration approvals between 1977 and 2007 reaches a directionally similar conclusion, finding that first-of-a-kind entrants spent 34% longer in regulatory approval than follow-on entrants and that this asymmetry discourages pioneering innovation in particular [84]. That study concerns a different jurisdiction, a different regulatory instrument and a period ending a decade before the MDR, and under the exclusion criteria set out in Section 2.2 it is admitted only as a non-EU comparator. It is not a third EU-relevant leg of the argument, and it is not counted as one here. The EU-specific evidence for the regulation-to-innovation link therefore rests on two sources, a calibrated structural model and a voluntary manufacturer survey, which agree in direction while differing in method; the older United States evidence shows only that the mechanism is not unique to the European framework.

For the framework, this is the clearest transmission from the Regulation node to the Innovation node that the literature supports: regulatory requirements set the evidence burden; the evidence burden lengthens and adds variance to the development path; the lengthened path erodes the appropriable return; and the erosion shifts both the level and the location of development effort. The consequence for European healthcare systems is second-order but real: if first launch migrates, European patients receive new technologies later, and European clinicians accumulate experience with them later, regardless of whether the device is eventually certified in the Union. What remains unresolved is the size of the effect in observed rather than calibrated data. The December 2025 proposal addresses assessment duration through statutory timelines and priority pathways but contains no patent-term compensation provision, so the specific mechanism identified by the model is left in place [12]. Whether the proposed timelines bind in practice, and whether notified-body capacity permits them, will determine how much of the modelled incentive loss the revision recovers, and that is an empirical question that can only be answered after 2028.

## 10. External Market Access: Trade, Procurement and Supply Resilience

The EU is a strong net exporter of medical technology, with exports rising faster than imports in recent years [85]. However, this trade surplus masks vulnerabilities: key segments such as respirators and disposables are heavily dependent on a few non-EU suppliers, while high-value equipment is primarily exported. Major partners include the US, China, Japan, and Mexico. Evolving procurement policies and concentrated import dependencies are driving the EU to rethink its sourcing and supply chain strategies to maintain its leadership and reduce risk exposure. Import dependency varies systematically by product category, but this review does not quantify it. A conventional approach to estimating import dependence for a defined product category can be calculated as follows:

(1) Import dependence = Imports/(Apparent domestic consumption) × 100%,

where

(2) Apparent domestic consumption = Production + Imports − Exports.

The composition of trade flows is as significant as the overall trade volumes. European firms typically design, final-assemble, and export high-value systems and implants, with substantial manufacturing operations in Germany, France, Switzerland, and northern Italy. These activities generate stable export streams and support robust service aftermarkets. In contrast, commoditised consumables, including certain catheter and dressing lines as well as a significant proportion of personal protective equipment and basic diagnostics, have increasingly been imported over the past decade from low-cost Asian suppliers, particularly manufacturers based in mainland China [86].

Driven by cost pressures, the EU has increasingly sourced disposable medical devices from Asia, with China supplying up to a third of imports in some categories [87]. This dependency creates vulnerabilities and has prompted the EU to adopt “de-risking” policies. In 2025, the European Commission excluded many Chinese suppliers from public tenders over €5 million to address market access concerns and reduce systemic risk [88]. Because public tenders dominate high-value device purchases, this policy shift is likely to redirect demand to European or trusted international suppliers. These actions show how trade and procurement rules now play a powerful role in shaping the EU medical device market.

Most finished medical devices face low tariffs in the EU, thanks to favourable schedules and trade agreements. However, non-tariff barriers, like complex customs rules, export licences, and extra documentation, pose greater risks for MedTech firms than tariffs themselves [89]. Delays from border checks or paperwork can disrupt manufacturing, especially for critical components. For the sector, customs procedures are less about taxes and more about avoiding costly logistical bottlenecks.

Brexit has created ongoing trade barriers for medical device companies, forcing many to duplicate regulatory filings or relocate manufacturing functions inside the EU to keep market access [90]. Some firms have reorganised supply chains to avoid customs and compliance hurdles, while others absorb higher costs to serve both the UK and EU. The UK’s changing regulations add more complexity, directly affecting manufacturing, warehousing, and distribution decisions across Europe.

Geopolitical tensions and export controls are reshaping Europe’s MedTech sector. COVID-19 exposed the dangers of relying on single suppliers, sparking a push for strategic autonomy in health goods through domestic production support, subsidies, and closer scrutiny of investments [91]. Tariffs and trade barriers now mean companies must favour resilient, diversified supply chains over the lowest cost. New EU industrial incentives and reshoring policies show governments are willing to back this shift. Logistics and customs efficiency are crucial, especially for SMEs, as delays can mean lost opportunities. EU efforts to modernise customs and digitalise certificates are positive steps, but inconsistent implementation and extra MDR/IVDR declarations still complicate cross-border trade [89]. Streamlined digital certification and better alignment between trade and regulatory processes are vital for Europe’s competitiveness.

EU free-trade agreements open global markets for European medical device manufacturers, driving export growth and competitiveness. However, when trading partners limit access to their markets, the EU must act to protect its own interests, as seen in recent restrictions on Chinese participation in public tenders. To succeed, European firms need continued access to key markets while the EU defends its strategic autonomy and supply chain security [85]. Effective trade and procurement policies are now crucial for the sector’s future. Trade and procurement policy therefore acts on the framework’s market-access node independently of conformity assessment.

Currency volatility and trade financing significantly impact exporters. Medical instrument companies invoicing in euros but paying suppliers in dollars or yuan face exchange rate risks that can cut into profits. To mitigate this, they rely on trade finance tools and hedging strategies. Smaller firms are particularly vulnerable due to limited access to advanced hedging and higher financing costs. Larger exporters benefit from credit insurance and government support, while micro exporters often depend on distributor pre-financing or local partnerships to reduce risk. Access to financing and effective currency risk management are crucial for winning long-term contracts and maintaining cross-border sales. The tender risk can be estimated with the use of using a weighted sum:

(3) $$R_{C}=\sum_{j=1}^{n}w_{j}X_{\mathrm{cj}},\;\sum_{j=1}^{n}w_{j}=1$$

where *R_c_* is the risk score for country *c*, *X_cj_* is the normalised score for risk factor *j,* and *w_j_* is its assigned weight. The normalised factor scores could range from 0 to 100.

**Figure 11:**
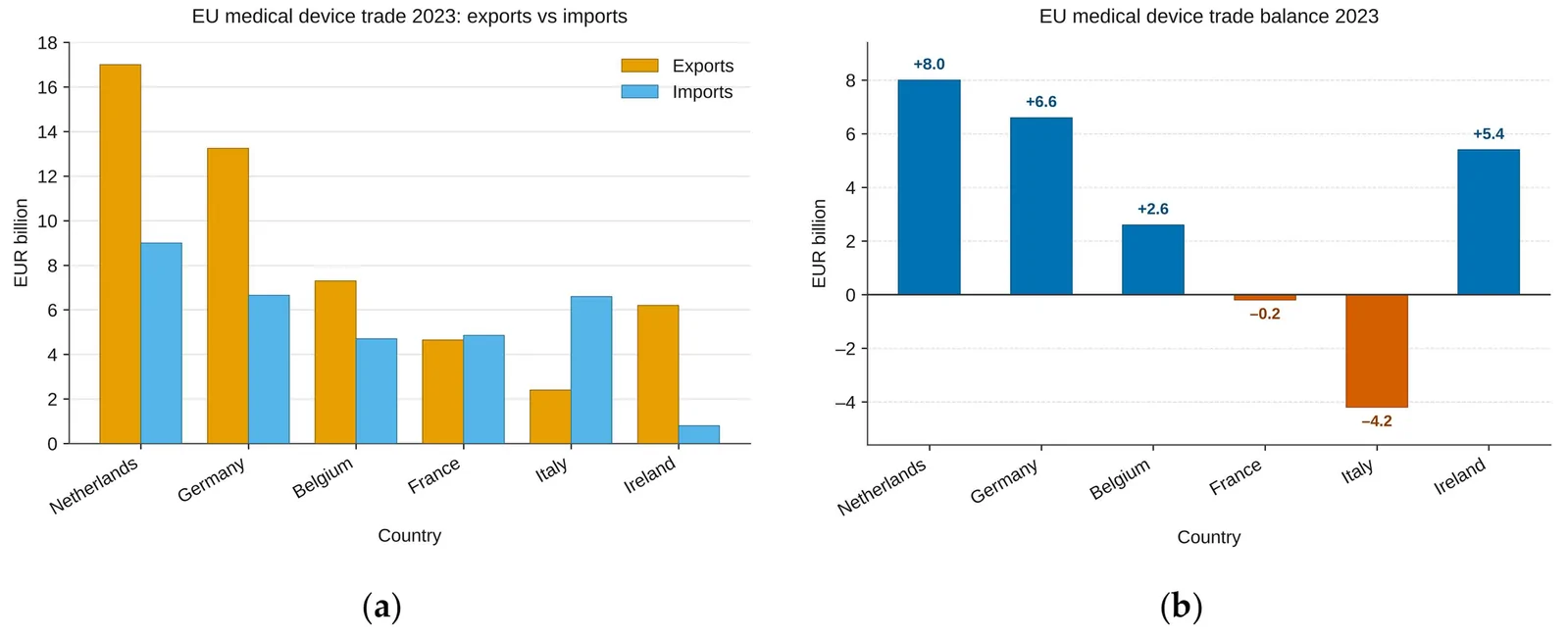
EU medical technology trade in 2023, extra-EU trade only (intra-EU flows excluded). (**a**) EU medical device exports and imports; (**b**) EU medical device trade balance.

Regulation and trade are closely linked in the EU medical device market. CE marking and compliance with MDR, IVDR, and EUDAMED requirements affect both EU market access and export opportunities, as many countries recognise the CE mark as a quality standard. Certification delays can limit exports and disrupt distributor relationships, while efficient, harmonised certification boosts the reputation and market reach of European manufacturers [92]. Trade and regulation influence each other, shaping both policy and market outcomes. Figure 11 shows EU medical technology trade figures.

Europe’s medical device market is shaped by trade dynamics, supply resilience, and regulatory requirements. While European exporters excel in high-value devices, dependence on external suppliers for components creates vulnerabilities. Recent policy changes and efforts to reduce single-country risk highlight the need for diversified supply chains and domestic manufacturing. Manufacturers should plan for tariffs, longer regulatory lead times, and stay engaged with policy developments to compete for public tenders. Companies that combine efficient manufacturing, fast regulatory compliance, and strong logistics will succeed in this evolving environment.

## 11. Quantitative Synthesis: Relationships Across the Framework

The purpose of this section is not to add further market values but to state in one place the relationships that the preceding sections identify between variables, and to grade the evidence behind each. Six relationships carry the argument.

• R1. Regulation to Evidence. Conformity assessment under the MDR and IVDR lengthened the application-to-certificate path to median totals of approximately 17–19 months for in vitro diagnostics and 19–22 months for medical devices, with pre-review and certificate issuance—neither of which is technical assessment—accounting for a substantial share of the total (Section 7). The phase medians and the reported totals are not arithmetically consistent for the MDR technical-documentation route, as set out in Section 7. Evidence grade: measured, from a repeated industry survey with disclosed methodology; not independently audited, self-selected in its respondent base, and internally inconsistent in one sub-sample. Independent verification becomes available from 1 January 2028, when Article 4 of Implementing Regulation (EU) 2026/977 requires every notified body to publish the median duration from application to certification [18].
• R2. Evidence to innovation (level). Longer and more variable assessment paths reduce the appropriable return on device development because patent life is consumed by review. Modelled effect: introducing a three-year supplementary protection certificate limited to Class III devices raises EU-directed development effort by 13.46% (95% CI 12.8–14.1%) in a single-market EU calibration, and total development effort by 5.6% (95% CI 4.3–7.1%) in the two-market setting. The figure is the effect of a hypothetical remedy under one calibration, not a measurement of loss already incurred [16]. Evidence grade: modelled, mechanism-identified, calibration-dependent; not validated against observed R&D expenditure.
• R3. Evidence to innovation (location). Manufacturers report reallocating first approval and launch outside the EU [17]. United States evidence from 1977 to 2007 shows pioneering entrants facing disproportionately long approvals relative to follow-on entrants, admitted here only as a non-EU comparator [84]. Evidence grade: one EU manufacturer survey, voluntary and self-selected, supported by a non-EU historical comparator; consistent in direction, imprecise in magnitude, and resting on a single EU-relevant source.
• R4. Evidence intensity, segment growth and market structure. At segment level, the association between evidence intensity (Table 1) and growth (Figure 4) is negative, not positive: the highest-burden segments grow most slowly. The better-supported claim concerns participation rather than growth—evidence requirements narrow the set of firms able to compete in the most regulated segments (Section 5). Evidence grade: observed association across heterogeneous commercial sources at segment level only, with no device-level dataset, no controlled comparison and no identification of causal direction; the participation claim is mechanistic and unquantified. This is the weakest of the six relationships on the innovation side of the chain.
• R5. Regulation and HTA to market access. Certification is necessary but not sufficient. From 2026, the joint clinical assessment applies a second, Union-level evidence filter to Class IIb and Class III devices and class D in vitro diagnostics that have passed expert-panel consultation, with the first two devices selected on 6 October 2026 and further selections expected quarterly [65]. Evidence grade: prospective; the mechanism is legally established and has begun to operate, but no assessment has yet concluded, and no outcome data exist.
• R6. Market access to healthcare-system impact. Product rationalisation, withdrawal of low-margin legacy devices and delayed entry translate into availability effects for providers and patients. Evidence grade: reported by manufacturers and acknowledged in the Commission’s own problem definition [12], and partially documented by the Commission’s study supporting the monitoring of the availability of medical devices on the EU market, which has run repeated notified-body and economic-operator surveys since 2023 and publishes results through a public dashboard [28]. That source is survey-based and aggregated: it records that discontinuations occur and that low-volume and legacy devices are most affected, but it does not provide device-level availability linkable to certification records, so the magnitude of the effect on provider-level availability remains unmeasured. This is the weakest link in the chain.

Table 2 reports the distribution of the retained corpus across the five framework dimensions and the evidence-type axis. The fragmentation asserted in the Introduction is visible in that distribution: a minority of retained documents carry a cross-cutting code, and fewer still link three or more dimensions. The distribution of evidence types is also markedly uneven across dimensions. The Regulation and Market Access dimensions are dominated by legal and policy analysis; the Innovation dimension by descriptive technology reporting, with a small and recent set of modelling contributions; and the Healthcare-System Impact dimension is the thinnest, containing almost no quantitative-empirical work on device availability at provider level.

Taken together, the six relationships describe a chain that is well evidenced at its regulatory origin, increasingly well evidenced at the innovation node, and poorly evidenced at its healthcare-system terminus. That asymmetry is itself a finding. The Union has built a regulatory regime whose stated justification is patient benefit, while the data infrastructure required to observe patient-level consequences, EUDAMED, whose transparency obligations became applicable only in May 2026 [15] is younger than the regime it is meant to render accountable. Until device-level availability data are published and linkable to certification records, R6 will remain an inference rather than a measurement, and the central policy question, whether the safety gained exceeds the access lost, will remain formally unanswerable.

## 12. Healthcare-System Implications, Limitations and Research Agenda

For manufacturers, the framework implies that regulatory strategy can no longer be a downstream function. Because conformity assessment and joint clinical assessment now impose partially overlapping but differently motivated evidence demands, and because the joint assessment operates on devices that have already passed expert-panel consultation, the clinical programme must be designed years in advance to satisfy both. The practical consequences are portfolio triage against certification hazard rather than margin alone; early, documented notified-body engagement; and treating post-market clinical follow-up as an asset that supports reimbursement rather than a compliance cost. For firms without internal capacity, the rational responses are partnership, licensing or acquisition, which is why the evidence requirement shows up in the data as market concentration.

For healthcare providers and payers, the implications are more ambivalent than either the regulatory or the commercial literature suggests. Stronger clinical evidence and Union-level assessment should improve the quality of adoption decisions and reduce duplicated national assessment effort. Against this, providers face a narrowing supplier base in several categories, the discontinuation of legacy devices whose recertification cannot be justified commercially, and capital-planning uncertainty when replacement equipment is subject to an unbounded certification path. Joint clinical assessment will not by itself resolve this: reimbursement remains national, contextualisation of Union-level assessment within national processes is unsettled, and HTA bodies themselves report uncertainty about timing and selection [52].

For patients, the consequences are concentrated where they are hardest to observe. If first launches migrate outside the Union [17], the effect is a lag in access to new technology that no register records, because a device never submitted generates no data. The clearest illustration is orphan devices: the Union has no legal orphan-device designation, no dedicated incentive and no harmonised assessment pathway, relying instead on non-binding guidance [93] and on Member State derogations under Article 59 of the MDR, an arrangement that comparative analysis characterises as flexibility interpreted rather than codified [36]. The December 2025 proposal would introduce orphan-device criteria with priority review [12], which is a material improvement; whether it is sufficient depends on incentives the proposal does not contain.

For policy, three implications follow directly from the graded relationships. First, procedure and capacity are not substitutes: statutory conformity-assessment timelines will bind only if notified-body throughput can meet them, and both the December 2025 proposal and Implementing Regulation (EU) 2026/977 address procedural harmonisation more directly than capacity [18]. Second, if the mechanism linking assessment duration to development incentives is correct, then time lost to review requires either shortening or compensation, and the current proposal chooses the first while declining the second; the economic case for a device supplementary protection certificate is therefore left open rather than settled [16]. Third, evaluation of the reform requires data that are only now beginning to exist in published form. The notified-body reporting duty under Article 4 of Implementing Regulation (EU) 2026/977, applicable from 1 January 2028, and the full activation of EUDAMED’s transparency modules are therefore preconditions for evidence-based policy rather than administrative details.

Five limitations qualify for these conclusions. The review is restricted to English-language sources, which may under-represent national regulatory and HTA literature published in other Union languages. For market magnitudes, it relies on commercial reports whose methodologies are not fully disclosed; these are used only as ranges and never as the sole basis for a claim, but the resulting figures carry wider uncertainty than their presentation in the source material suggests. The heterogeneity of outcome measures precludes meta-analysis, so the synthesis grades evidence rather than pooling it. And the review closes at a discontinuity: the legislative revision is before the co-legislators, joint clinical assessment of devices has only begun, and EUDAMED obligations have only just taken effect, so the findings constitute a baseline against which the reform can be assessed rather than an assessment of it. A fifth limitation concerns the indicators: the segment evidence-intensity classification in Table 1 is a rule-based mapping from the classification criteria of Annex VIII of the two Regulations and records the predominant case within each segment, so it does not capture the variation in risk class within a segment, which is precisely the resolution at which the regulatory burden actually operates.

The research agenda follows from the weakest links. Priority should be given to: (i) construction of a device-level dataset linking EUDAMED certification records to national availability and procurement data, which would convert R6 from inference to measurement and would extend the aggregated, survey-based coverage of the Commission’s availability study [28]; (ii) empirical validation of the modelled innovation effect against observed R&D expenditure and filing behaviour, exploiting the 2026–2028 reform as a natural experiment; (iii) measurement of the devices that were never submitted or were withdrawn, which is the counterfactual the entire debate presupposes and none of the literature observes; (iv) use of the notified-body duration and cost series published from 2028 under Article 4 of Implementing Regulation (EU) 2026/977 to test, for the first time with independent data, whether the variance of certification duration behaves as the mean does, and whether the maximum timelines bind; (v) evaluation of whether joint clinical assessment reduces or merely relocates duplicated national assessment effort; and (vi) analysis of whether the breakthrough and orphan pathways, once operative, change the composition of devices entering the Union rather than only their speed.

## 13. Conclusions

This review aimed to replace parallel descriptions of the European medical device sector with an integrated account of how its parts interact. The five-stage framework: Regulation, Evidence, Innovation, Market Access, and Healthcare-System Impact, was used both as an analytical structure and as the coding scheme for a reproducible synthesis of the 2017–2026 literature. Its principal result is that the effects commonly attributed to the MDR and the IVDR are better understood as transmission through a chain than as a single regulatory shock. Evidence requirements, not legal text, are the operative variable; certification duration and its variance, not compliance cost alone, are the channel through which those requirements reach innovation; and health technology assessment and procurement, not certification, determine whether an approved device is actually used.

The quantitative synthesis shows this chain to be unevenly evidenced. Certification duration is measured, if only through industry self-report: median application-to-certificate paths of approximately 17–19 months for in vitro diagnostics and 19–22 months for medical devices, with a large share consumed by phases that are not technical assessment. The innovation consequence has moved from assertion to estimation, with a two-market model estimating that a three-year Class III supplementary protection certificate would raise EU-directed development effort by 13.46% in a single-market calibration and total effort by 5.6% in the two-market setting, and manufacturer survey evidence recording a shift in first launches away from the Union. The healthcare-system consequence remains the least measured link: the Commission’s availability study documents that discontinuations occur but only in aggregated, survey-based form; no dataset reports device availability at provider level, and the infrastructure that could, EUDAMED, became mandatory only in 2026. The market itself, at €131–170 billion in 2024 and growing at 4–6%, conceals segment rates that are inversely rather than positively associated with evidence intensity: the most regulated segments grow most slowly, and the clearer effect of the evidence requirement is compositional, determining which firms can participate in those segments rather than how fast the segments expand.

The period covered by this review closes at a discontinuity rather than at a settled state. The Commission’s targeted revision of December 2025 remains before the Parliament and the Council, and its proposed disapplication of the Artificial Intelligence Act to devices has already been rejected in the Digital Omnibus; Implementing Regulation (EU) 2026/977 has nonetheless made maximum conformity-assessment timelines binding from February 2027 and notified-body duration reporting mandatory from 2028; the first two devices were selected for joint clinical assessment in October 2026; and EUDAMED’s transparency obligations have only just taken effect. The framework and the synthesis presented here are therefore offered as a baseline against which those reforms can be evaluated, and as an argument for the kind of evaluation that should be undertaken: not whether the rules changed, which is not in dispute, but what measurable consequences the change produces for innovation, for market entry, and for the healthcare systems the rules exist to serve.

## Figures and Tables

**Table 2:** Distribution of the retained corpus across the framework dimensions and evidence types. Documents may carry more than one dimension code, so column totals exceed the number of retained documents. Full coded dataset in Supplementary Material S3.

Framework Dimension	Documents Coded	Cross-Cutting	Dominant Evidence Type
Regulation	31	15	Legal or policy analysis
Evidence	7	6	Quantitative-empirical (survey)
Innovation	27	17	Descriptive or market reporting
Market Access	41	16	Legal or policy analysis
Healthcare-System Impact	19	12	Qualitative-empirical
Total retained	91	32	—

## Data Availability

The datasets created by the review (screening log, included-document list, coded corpus) are available in the Supplementary Materials.

## Supplementary Materials

The following supporting information can be downloaded at https://www.mdpi.com/article/10.3390/healthcare14213822/s1. S1:PRISMA 2020 checklist. S2: PRISMA 2020 flow diagram. S3: Included documents and coding. S4: Screening log, scope, procedure and status.

## Abbreviations

The following abbreviations are used in this manuscript:

BtX	Breakthrough Devices
CAGR	Compound Annual Growth Rate
CER	Clinical Evaluation Report
EEA	European Economic Area
EUDAMED	European Database on Medical Devices
EU	European Union
HTA	Health Technology Assessment
HTAR	Health Technology Assessment Regulation
IVD	In Vitro Diagnostics
IVDR	In Vitro Diagnostic Regulation
JCA	Joint Clinical Assessment
JSC	Joint Scientific Consultation
MDCG	Medical Device Coordination Group
MDR	Medical Device Regulation
NB	Notified Body
NCAs	National Competent Authorities
PMCF	Post-Market Clinical Follow-up
PMS	Post-Market Surveillance
POC	Point-of-Care
POCT	Point-of-Care Testing
PRISMA	Preferred Reporting Items for Systematic Reviews and Meta-Analyses
QMS	Quality Management System
RWE	Real-World Evidence
SaMD	Software as a Medical Device
SMEs	Small and Medium-sized Enterprises
SPC	Supplementary Protection Certificate
TDA	Technical Documentation Assessment
UDI	Unique Device Identification
