# Supplementary material for: The European Medical Device Ecosystem: Interactions Between Regulation, Innovation, Market Access, and Healthcare Systems
Source: Healthcare (Basel). 2026 Nov 9;14(21):3822. doi: 10.3390/healthcare14213822 (PMC13763232; doi:10.3390/healthcare14213822)
Supplement: Supplementary file 1 [file healthcare-14-03822-s001.zip › healthcare-4613562-supplementary.pdf]

Supplementary Material S1 (healthcare-4613562)

PRISMA 2020 checklist

Manuscript title: The European Medical Device Ecosystem: Interactions Between Regulation, Innovation, Market Access, and Healthcare Systems

This review is a structured, protocol-driven synthesis. The checklist is completed in full and, where an item does not apply, the reference says so and the reason is given in the notes below rather than left blank.

Section and topic	Item #	Checklist item	Location where reported, or reason not reported
**TITLE**	1	Title: identify the report as a systematic review.	Title page. Reported as a structured review.
**ABSTRACT**	2	Abstract: see the PRISMA 2020 for Abstracts checklist.	Abstract. Partially reported.
**INTRODUCTION**	3	Rationale: describe the rationale for the review in the context of existing knowledge.	Section 1, paragraphs 3–5.
	4	Objectives: provide an explicit statement of the objective(s) or question(s) the review addresses.	Section 1, paragraph 6 (four research questions).
**METHODS**	5	Eligibility criteria: specify the inclusion and exclusion criteria and how studies were grouped for the syntheses.	Section 2.2, 'Inclusion criteria', 'Exclusion criteria', 'Geographical definition of the EU', 'Document types'; grouping in Section 2.2, final paragraph (three-axis coding).
	6	Information sources: specify all databases, registers, websites, organisations and other sources searched, and the date when each was last searched.	Section 2.2, 'Databases searched' and 'Search period'.
	7	Search strategy: present the full search strategies for all databases, registers and websites, including any filters and limits used.	Section 2.2, 'Search strings'. Full per-database strings with filters and hit counts in S4.
	8	Selection process: specify the methods used to decide whether a study met the inclusion criteria, including how many reviewers screened each record and whether they worked independently.	Section 2.2, 'Screening process'.
	9	Data collection process: specify the methods used to collect data from reports, including how many authors collected data and whether they worked independently.	Section 2.2, final paragraph; coded dataset in S3.
	10a	Data items: list and define all outcomes for which data were sought.	Section 2.3 and Section 11 (certification duration, development effort, segment growth, market concentration).
	10b	Data items: list and define all other variables for which data were sought.	Section 2.2, final paragraph (three coding axes); Section 5, Table 1.
	11	Study risk of bias assessment: specify the methods used to assess risk of bias in the included studies.	Not applicable. Source-level reliability is instead reported as the evidence grade attached to each relationship in Section 11.
	12	Effect measures: specify for each outcome the effect measure(s) used in the synthesis or presentation of results.	Not applicable. Outcome measures are heterogeneous and are reported in their original units.
	13a	Synthesis methods: describe the processes used to decide which studies were eligible for each synthesis.	Section 2.2, final paragraph (coding against framework dimensions determines which documents inform which relationship).
	13b	Synthesis methods: describe any methods required to prepare the data for presentation or synthesis.	Section 2.3 (four rules: range reporting, currency conversion, scenario presentation, indicator construction).
	13c	Synthesis methods: describe any methods used to tabulate or visually display results.	Section 2.3; Table 1 (Section 5); Table 2 (Section 11);

Section and topic	Item #	Checklist item	Location where reported, or reason not reported
			figure captions state sources and plotting software.
	13d	Synthesis methods: describe any methods used to synthesise results and provide a rationale for the choice(s).	Section 2.1 (five-dimension framework) and Section 11 (six relationships with evidence grades). Narrative synthesis; no statistical pooling, for the reason given in Section 2.2, paragraph 1.
	13e	Synthesis methods: describe any methods used to explore possible causes of heterogeneity among study results.	Section 2.3, rule 1 (scope of each estimate stated); Section 5 (segment definition differences between providers).
	13f	Synthesis methods: describe any sensitivity analyses conducted.	Not conducted. Currency-conversion sensitivity is acknowledged qualitatively in Section 2.3, rule 2.
	14	Reporting bias assessment: describe any methods used to assess risk of bias due to missing results in a synthesis.	Not formally assessed. Self-selection in the industry survey underlying R1 is stated in Section 7 and in the grading of R1.
	15	Certainty assessment: describe any methods used to assess certainty in the body of evidence for an outcome.	Section 2.1, final paragraph, and Section 11: each relationship carries an explicit evidence grade (measured/modelled / survey / prospective / inferred).
**RESULTS**	16a	Study selection: describe the results of the search and selection process, ideally using a flow diagram.	Section 2.2, 'Records identified and retained'; flow diagram in S2.
	16b	Study selection: cite studies that might appear to meet the inclusion criteria but were excluded and explain why.	S2, S4 (screening log, exclusion reasons at full-text stage).
	17	Study characteristics: cite each included study and present its characteristics.	S3 (all included documents with document type, evidence type, device domain and framework dimension codes).
	18	Risk of bias in studies: present assessments of risk of bias for each included study.	Not applicable.
	19	Results of individual studies: present summary statistics and effect estimates for each study.	Not applicable. Individual quantitative results are reported in the relevant sections in their original units.
	20a	Results of syntheses: briefly summarise the characteristics and risk of bias among contributing studies.	Section 11, Table 2 (distribution of the corpus across dimensions and evidence types).
	20b	Results of syntheses: present results of all statistical syntheses conducted.	Not applicable. No statistical synthesis was conducted.
	20c	Results of syntheses: present results of all investigations of possible causes of heterogeneity.	Section 5 (segment scope differences); Section 7 (inconsistency between phase medians and reported totals).
	20d	Results of syntheses: present results of all sensitivity analyses.	Not applicable.
	21	Reporting biases: present assessments of risk of bias due to missing results for each synthesis assessed.	Not formally assessed.
	22	Certainty of evidence: present assessments of certainty in the body of evidence for each outcome assessed.	Section 11, evidence grade attached to each of R1–R6.
**DISCUSSION**	23a	Provide a general interpretation of the results in the context of other evidence.	Section 11, final paragraph; Section 13.

Section and topic	Item #	Checklist item	Location where reported, or reason not reported
	23b	Discuss any limitations of the evidence included in the review.	Section 12, limitations paragraph; evidence grades in Section 11.
	23c	Discuss any limitations of the review processes used.	Section 12, limitations paragraph (five limitations, including language restriction, commercial sources, absence of meta-analysis and the resolution of the Table 1 classification).
	23d	Discuss implications of the results for practice, policy and future research.	Section 12 (manufacturers, providers and payers, patients, policy) and research agenda.
**OTHER INFORMATION**	24a	Registration and protocol: provide registration information, including the register name and registration number, or state that the review was not registered.	Not registered.
	24b	Indicate where the review protocol can be accessed, or state that a protocol was not prepared.	Protocol not prospectively registered. The operative protocol is reported in Section 2.2.
	24c	Describe and explain any amendments to information provided at registration or in the protocol.	Section 2.2, 'Search period': targeted update of legal and institutional sources to 10 October 2026, with the reason stated.
	25	Support: describe sources of financial or non-financial support for the review, and the role of the funders.	Funding statement in the back matter. The funder had no role in the design, conduct or reporting of the review.
	26	Competing interests: declare any competing interests of review authors.	Conflicts of Interest statement in the back matter.
	27	Availability of data, code and other materials: report which of the following are publicly available and where: data collection form; data extracted; data used for analyses; analytic code; other materials.	Data Availability Statement in the back matter; S3 (coded corpus) and S4 (screening log). Figures were produced in Python 3.11 with Matplotlib 3.8.

Supplementary Material S2 (healthcare-4613562)

PRISMA 2020 flow diagram

Manuscript title: The European Medical Device Ecosystem: Interactions Between Regulation, Innovation, Market Access, and Healthcare Systems

Search period: 1 January 2017 to 30 June 2026, with a targeted update of legal and institutional sources to 10 October 2026. Geographical scope: EU-27 and EEA, with Switzerland, the United Kingdom and the United States admitted only as explicit comparators.

**Identification**	**Records identified from databases**<br>Scopus (n = 2434)<br>Web of Science Core Collection (n = 1310)<br>PubMed / MEDLINE (n = 1459)<br>ScienceDirect (n = 1023)<br>Total (n = 6226)	**Records identified from other sources**<br>EUR-Lex, European Commission DG SANTE, MDCG guidance repository, EUDAMED documentation, Eurostat, OECD, MedTech Europe<br>Total (n = 500)
	↓	
	**Records after duplicates removed**<br>(n = 1997)	**Duplicate records removed**<br>(n = 4729)
	↓	
**Screening**	**Records screened on title and abstract**<br>(n = 1997)	**Records excluded at title and abstract**<br>(n = 1706)
	↓	
	**Full-text documents assessed for eligibility**<br>(n = 291)	**Full-text documents excluded, with reasons**<br>No EU/EEA dimension and not an admitted comparator (n = 28)<br>Single-device clinical study with no regulatory, economic or system-level dimension (n = 33)<br>No verifiable data source (vendor material, commentary) (n = 41)<br>Duplicate reporting of the same underlying dataset (n = 25)<br>Abstract only retrievable (n = 33)<br>Outside the search period (n = 5)<br>Not in English (n = 4)<br>Other (n = 29)
	↓	
**Included**	**Documents included in the synthesis**<br>Total (n = 93)<br>Peer-reviewed articles and reviews (n = 41)<br>EU legislation and legislative proposals (n = 5)<br>Regulatory guidance and competent-authority documents (n = 17)<br>Official statistics (n = 6)<br>Trade-association surveys and position papers (n = 4)<br>Commercial market and company reports (n = 15)<br>Company report (n = 3)<br>Research report (n = 2)	

Supplementary Material S3 (healthcare-4613562)

Included documents and coding

Manuscript title: The European Medical Device Ecosystem: Interactions Between Regulation, Innovation, Market Access, and Healthcare Systems

The table lists the 93 documents in the revised manuscript's reference list, each coded on three axes. Framework dimension codes are abbreviated REG (Regulation), EVI (Evidence), INN (Innovation), MA (Market Access) and HSI (Healthcare-System Impact). Rows shaded in the dimension columns carry two or more codes and are therefore counted as cross-cutting.

No.	Reference (abbreviated)	Document type	Evidence type	Device domain	Framework dimension(s)	No. of dimensions	Note
1	Garzotto, F.; Comoretto, R.I.; Dorigo, L.; Gregori, D.; Zotti, A.; Meneghesso, G.; Gerosa, G.; Bonin, M. Preparing Healthcare, Academic Institutions, and Notified Bodies for Their Involvement in the…	Peer-reviewed	Legal/policy analysis	Cross-domain	REG, INN	2	
2	Universal care under strain: navigating population ageing in Europe	Official statistics	Quantitative-empirical	Cross-domain	HIS	1	
3	OECD Full Report: Health at a Glance: Europe 2024 - State of Health in the EU Cycle; 2024;	Official statistics	Quantitative-empirical	Cross-domain	HSI	1	
4	Factsheet for Manufacturers of Medical Devices - Public Health	Regulatory guidance	Legal/policy analysis	Cross-domain	REG	1	
5	Kahles, A.; Volckmar, A.-L.; Goldschmid, H.; Salto-Tellez, M.; Vogeser, M.; Stone, J.; Brüggemann, M.; Budczies, J.; Kazdal, D.; Salgado, R.; et al. Regulation of Laboratory-Developed Tests and in-H…	Peer-reviewed	Legal/policy analysis	In vitro diagnostics	REG, EVI	2	
6	Choe, M.; Shim, J.H.; Heo, C.Y. A Comparative Study of Regulatory Perspectives on Innovative Medical Devices in Korea and the United States. Expert Review of Medical Devices 2022, 19, 229–235, doi:1…	Peer-reviewed	Legal/policy analysis	Cross-domain	REG, INN	2	*Korea and US; admitted under exclusion criterion (c) as comparator.*
7	Tichiwangana, T.R.; Ji, Q.; Fan, X.; Ying, T.; Chen, X. Revolutionizing Breast Cancer Care: The Synergy of AI-Powered Diagnostics, Haptic-Based Biopsy Simulators, and Advanced Surgical Techniques. E…	Peer-reviewed	Descriptive	Software / digital health	INN	1	
8	Ahsan, Z.; Mahmud, S.; Chowdhury, M.R.; Ali, Y.; Fuad, T.B.; Islam, S.; Alam, Z.F.; Saha, S.; Islam, Md.A.; Hossain, N. Advances in Additive Manufacturing for Medical Robotics: A Review. Results in …	Peer-reviewed	Descriptive	Implantable devices	INN	1	
9	Sallam Ma'aitah, M.K.; Idoko, J.B.; Yaseen, B.A. VitalGuard-AI: A Real-Time Multi-Modal Deep Learning Framework for Intelligent Health Monitoring Using Wearable IoT Devices. Biomedical Signal Proces…	Peer-reviewed	Quantitative-empirical	Software / digital health	INN	1	
10	Heymer, E.J.; Clark, S.A.; Campbell, H.; Ribeiro, S.; Fox, A.; Lucidarme, J.; Bai, X.; Ladhani, S.N.; Borrow, R. Impact of Molecular Point-of-Care Testing on Surveillance and Public Health Managemen…	Peer-reviewed	Quantitative-empirical	In vitro diagnostics	INN, HSI	2	
11	Robinson, A.V.; Noël, J.; Peckham-Cooper, A.; Pegna, V. How Can the Model for a Sustainable Surgical Pathway Be Enhanced by Digital Medicine? Surgery (Oxford) 2025, 43, 153–159, doi:10.1016/j.mpsur.…	Peer-reviewed	Descriptive	Surgical instruments / robotics	INN, HSI	2	
12	European Parliament. Document 52025PC1023. Proposal for a REGULATION OF THE EUROPEAN PARLIAMENT AND OF	EU legislation	Legal/policy analysis	Cross-domain	REG	1	

No.	Reference (abbreviated)	Document type	Evidence type	Device domain	Framework dimension(s)	No. of dimensions	Note
	THE COUNCIL Amending Regulations (EU) 2017/745 and (EU) 2017/746; 2025;						
13	MedTech Europe Revision Proposal Is First Step towards Fixing Europe’s Complex Medical Devices & Diagnostics Rules. MedTech Europe 2025.	Trade association	Descriptive	Cross-domain	REG, MA	2	
14	2026 Work Programme - Member State Coordination Group on HTA (HTACG) - Public Health	Regulatory guidance	Legal/policy analysis	Cross-domain	MA	1	
15	European Commission. Public Health. EUDAMED	Regulatory guidance	Legal/policy analysis	Cross-domain	REG, HSI	2	
16	Peters, A. Regulatory Delays and Patent Term Erosion in Medical Device Innovation: A Two-Market Analysis of EU and US Frameworks. Eur J Law Econ 2026, 62, 131–164, doi:10.1007/s10657-026-09873-3.	Peer-reviewed	Modelling / economic	Cross-domain	REG, INN	2	*Primary source for R2.*
17	McDermott, O.; Folan, M.; Stafylas, P. An Impact Assessment of the European Medical Device Regulations Implementation – the Current Status Quo. Expert Review of Medical Devices 2026, 23, 1137–1148, …	Peer-reviewed	Quantitative-empirical	Cross-domain	REG, INN	2	*Primary source for R3.*
18	European Commission Commission Implementing Regulation (EU) 2026/977 of 4 May 2026 Laying down Certain Uniform Quality Management and Procedural Requirements for the Conformity Assessment Activities…	EU legislation	Legal/policy analysis	Cross-domain	REG, EVI	2	*Implementing Regulation (EU) 2026/977. Earlier duplication resolved.*
19	European Parliament. Amending Regulations (EU) 2024/1689, (EU) 2018/1139 and (EU) 2023/1230 as Regards the Simplification of the Implementation of Harmonised Rules on Artifici…	EU legislation	Legal/policy analysis	Software / digital health	REG, INN	2	*Digital Omnibus.*
20	European Commission Joint Clinical Assessments for Medicinal Products - Public Health	Regulatory guidance	Legal/policy analysis	Cross-domain	MA	1	
21	Page, M.J.; McKenzie, J.E.; Bossuyt, P.M.; Boutron, I.; Hoffmann, T.C.; Mulrow, C.D.; Shamseer, L.; Tetzlaff, J.M.; Akl, E.A.; Brennan, S.E.; et al. The PRISMA 2020 Statement: An Updated Guideline f…	Peer-reviewed	Method guideline	Cross-domain	—	—	*Methodological reference.*
22	Inside the European Medtech Market: Insights and Regional Dynamics - Life Science Intelligence	Commercial market report	Descriptive	Cross-domain	MA	1	
23	MedTech Europe, from Diagnosis to Cure	Trade association	Descriptive	Cross-domain	MA	1	
24	Europe Medical Devices Market Size, Share & Report [2032]	Commercial market report	Descriptive	Cross-domain	MA	1	
25	Medical Devices Market Consulting and Research Reports	Commercial market report	Descriptive	Cross-domain	MA	1	
26	The Medical Technology Industry in Germany	Commercial market report	Descriptive	Cross-domain	MA	1	
27	Overview - Public Health - European Commission	Regulatory guidance	Legal/policy analysis	Cross-domain	REG	1	
28	European Commission, DG SANTE / HaDEA Study Supporting the Monitoring of Availability of Medical Devices on the EU Market - Public Health	Research report	Quantitative-empirical	Cross-domain	MA, HSI	2	*Primary source for the regrading of R6.*
29	European Medicines Agency Medical Device Legislation: Regulation (EU) 2017/745, Regulation (EU) 2017/746	Regulatory guidance	Legal/policy analysis	Cross-domain	REG	1	
30	European Commission Overview and Guidance on Medical Devices (MDR/IVDR)	Regulatory guidance	Legal/policy analysis	Cross-domain	REG	1	

No.	Reference (abbreviated)	Document type	Evidence type	Device domain	Framework dimension(s)	No. of dimensions	Note
31	European Commission. EU Contact Points of National Authorities MDR - IVDR	Regulatory guidance	Legal/policy analysis	Cross-domain	REG	1	
32	Medical Devices - Bulgarian Drug Agency	Regulatory guidance	Legal/policy analysis	Cross-domain	REG	1	
33	Hathout, L.; Shin, Y.E.; Rojanasarot, S.; Ezekekwu, E.; Vannan, D.; Folkert, M.R. Real-World Medical Device Reports of SpaceOAR Hydrogel Spacer: Analysis of the Food and Drug Administration Manufact…	Peer-reviewed	Quantitative-empirical	Implantable devices	EVI, HSI	2	*US FDA data; comparator only.*
34	HALMED - Agency for Medicinal Products and Medical Devices	Regulatory guidance	Legal/policy analysis	Cross-domain	REG	1	
35	European Commission, Medical Device Coordination Group Guidance on Breakthrough Devices (BtX) under Regulations 2017/745 & 2017/746 2025.	Regulatory guidance	Legal/policy analysis	Cross-domain	REG, INN	2	*MDCG 2025-9.*
36	Akodad, S.; Haon, B.; Rochaix, L.; Stevens, H. Orphan Medical Devices: Addressing the Regulatory and Access Gaps in the EU and US. Front. Public Health 2026, 13, doi:10.3389/fpubh.2025.1729821.	Peer-reviewed	Legal/policy analysis	Cross-domain	REG, MA, HSI	3	
37	Regulation (EU) 2023/607 of the European Parliament and of the Council of 15 March 2023 Amending Regulations (EU) 2017/745 and (EU) 2017/746 as Regards the Transitional Provisions for Certain Medica…	EU legislation	Legal/policy analysis	Cross-domain	REG	1	
38	Hamilton, L.; Goldman, E.; Dehipawala, S.; O'Hara, M. OP16 Environmental Sustainability Success Stories From the Pharmaceutical and Medtech Industries in Europe: A Targeted Literature Review. Value …	Peer-reviewed	Descriptive	Cross-domain	HSI	1	*Conference abstract.*
39	Data Bridge Market Research. Wearable and IoMT Medical Devices Market – Global Market Size, Share and Trends Analysis Report – Industry Overview and Forecast to 2032; 2024;	Commercial market report	Descriptive	Software / digital health	INN, MA	2	
40	Market Data Forecast Medical Devices Research Reports and Industry Overview	Commercial market report	Descriptive	Cross-domain	MA	1	
41	Abächerli, R. MedTech AI Regulation in Europe Compared to the USA. Journal of Electrocardiology 2024, 84, 15, doi:10.1016/j.jelectrocard.2024.05.030.	Peer-reviewed	Legal/policy analysis	Software / digital health	REG, INN	2	
42	Market Research Reports for Medical Devices and Diagnostics Sector	Commercial market report	Descriptive	In vitro diagnostics	MA	1	
43	Medical Devices Market Size, Demand, Analysis Report 2025 – 2030	Commercial market report	Descriptive	Cross-domain	MA	1	
44	Agrawal, V. Infectious Disease Molecular Diagnostics Market 2034	Commercial market report	Descriptive	In vitro diagnostics	INN, MA	2	
45	Siemens Healthineers Achieves Targets for 2024 Fiscal Year with Strong Fourth Quarter	Company report	Descriptive	Diagnostic imaging	MA	1	*Corporate press release; triangulation only.*
46	GLOBAL SURGICAL ROBOTICS MARKET 2024-2032	Commercial market report	Descriptive	Surgical instruments / robotics	INN, MA	2	
47	Microsurgery Market Size, Share and Growth Report [2032]	Commercial market report	Descriptive	Surgical instruments / robotics	MA	1	
48	MedTech Europe. The European Medical Technology in Figures	Trade association	Descriptive	Cross-domain	INN, MA	2	
49	The Historical Evolution of European Medical Device Regulation: From Fragmented National Systems to a Harmonized Supranational Framework. PHARMACIA 2026, 73, 1–10, doi:10.3897/pharmacia.73.e185464.	Peer-reviewed	Legal/policy analysis	Cross-domain	REG	1	

No.	Reference (abbreviated)	Document type	Evidence type	Device domain	Framework dimension(s)	No. of dimensions	Note
50	Eurostat. Population Structure and Ageing	Official statistics	Quantitative-empirical	Cross-domain	HSI	1	
51	Hub of The Chronic Noncommunicable Disease Network. Dental Abstracts 2026, 71, 39–41, doi:10.1016/j.denabs.2025.12.022.	Peer-reviewed	Descriptive	Cross-domain	HSI	1	*Dental Abstracts. Relevance to the cited claim is doubtful - review or replace.*
52	Healthcare Expenditure Statistics by Function, Provider and Financing Scheme	Official statistics	Quantitative-empirical	Cross-domain	HSI	1	
53	OECD. Full Report: Health at a Glance 2023	Official statistics	Quantitative-empirical	Cross-domain	HSI	1	*Different edition from entry 3; check which claim each supports.*
54	Medtech Industry Trends in 2024 and Beyond	Commercial market report	Descriptive	Cross-domain	INN, MA	2	
55	Seddon, J.J.M.; Currie, W.L. Cloud Computing and Trans-Border Health Data: Unpacking U.S. and EU Healthcare Regulation and Compliance. Health Policy and Technology 2013, 2, 229–241, doi:10.1016/j.hl…	Peer-reviewed	Legal/policy analysis	Software / digital health	REG	1	*Published 2013, before the stated search window; found via cross-references*
56	European Commission. Public Health. Manufacturers MD.	Regulatory guidance	Legal/policy analysis	Single-use / consumables	REG	1	
57	Pannonhalmi, Á.; Sipos, B.; Kurucz, R.I.; Katona, G.; Kemény, L.; Csóka, I. Advancing Regulatory Oversight of Medical Device Trials to Align with Clinical Drug Standards in the European Union. Pharm…	Peer-reviewed	Legal/policy analysis	Cross-domain	REG, EVI	2	
58	Zolghadri, M.; Besbes, M.; Bourgeois, V.; Saad, E. Micro-Electronic Chips Shortages and Obsolescence: An Empirical Study. Procedia CIRP 2023, 120, 1570–1575, doi:10.1016/j.procir.2023.09.216.	Peer-reviewed	Quantitative-empirical	Cross-domain	MA, HSI	2	
59	Mohammad, W.; Elomri, A.; Kerbache, L. The Global Semiconductor Chip Shortage: Causes, Implications, and Potential Remedies. IFAC-PapersOnLine 2022, 55, 476–483, doi:10.1016/j.ifacol.2022.09.439.	Peer-reviewed	Descriptive	Cross-domain	MA, HSI	2	
60	Ostermann, M.; Mathias, R.; Jahed, F.; Parker, M.B.; Hudson, F.D.; Harding, W.C.; Gilbert, S.; Freyer, O. Cybersecurity Requirements for Medical Devices in the EU and US - A Comparison and Gap Analy…	Peer-reviewed	Legal/policy analysis	Software / digital health	REG	1	
61	Alshaikh, R.A.; Walsh, K.A.; Spillane, S.; Harrington, P.; O'Neill, M.; Teljeur, C.; Ryan, M.; O'Driscoll, C.M. The Early Experiences of Health Technology Assessment Bodies in the Implementation of the European Union Health Technology Assessment Regulation for High-Risk Medical Devices: A Qualitative Study	Peer-reviewed	Qualitative-empirical	Cross-domain	EVI, MA	2	*Primary source for the HTA contextualisation claim.*
62	European Commission. The European Green Deal	Regulatory guidance	Legal/policy analysis	Cross-domain	REG	1	
63	Larkin, J.; Mulinari, S.; Ozieranski, P.; Lynch, K.; Fahey, T.; Ozaki, A.; Moriarty, F. Payments to Healthcare Organisations Reported by the Medical Device Industry in Europe from 2017 to 2019: An O…	Peer-reviewed	Quantitative-empirical	Cross-domain	MA	1	
64	IVDR & MDR Survey Results 2024. Public Report. MedTech Europe.	Trade association	Quantitative-empirical	Cross-domain	EVI	1	*Primary source for R1 (conformity-assessment durations).*
65	Regulation (EU) 2021/2282 of the European Parliament and of the Council of 15 December 2021 on Health Technology Assessment and Amending Directive 2011/24/EU (Text with EEA Relevance); 2021; Vol. 45…	EU legislation	Legal/policy analysis	Cross-domain	MA	1	*HTA Regulation.*
66	Keaney, J. Roche Sustainability Report; 2024;	Company report	Descriptive	In vitro diagnostics	MA	1	

No.	Reference (abbreviated)	Document type	Evidence type	Device domain	Framework dimension(s)	No. of dimensions	Note
67	Market Share of Leading Medical Device Players in Diagnostic Imaging in Europe 2023	Commercial market report	Descriptive	Diagnostic imaging	MA	1	*Source is a LinkedIn company page; replace with the underlying GlobalData report.*
68	Stryker Comprehensive Report 2024: Financial, Environmental, Social and Governance; 2024;	Company report	Descriptive	Implantable devices	MA	1	
69	Horn, D.; Sacarny, A.; Zhou, A. Technology Adoption and Market Allocation: The Case of Robotic Surgery. Journal of Health Economics 2022, 86, 102672, doi:10.1016/j.jhealeco.2022.102672.	Peer-reviewed	Quantitative-empirical	Surgical instruments / robotics	MA, HSI	2	*US data; comparator only.*
70	MedTech Dive. Top 10 Medtech Deals of 2024	Commercial market report	Descriptive	Cross-domain	MA	1	
71	Wang, L.; Parolin, E.; Chopra, Z.; Strohbehn, G.W. Oncology Pharmaceutical Mergers and Acquisitions Activity in the Precision Medicine Era. Drug Discovery Today 2026, 31, 104578, doi:10.1016/j.drudi…	Peer-reviewed	Descriptive	Cross-domain	—	—	
72	Boland, L.; Hopkinson, D.; Foxon, G. HTA189 Alignment of EU HTA and Member State Clinical Assessments and the Potential Impact on Value Perception. Value in Health 2024, 27, S391, doi:10.1016/j.jval…	Peer-reviewed	Descriptive	Cross-domain	MA	1	*Conference abstract.*
73	Krämer, A.; Franco, C.; Chamorro, I.P.; Bogdanov, G. International Markets and Developments for Medical Devices. In Medical Device Management; Springer Series in Healthcare Management and Innovation…	Peer-reviewed	Descriptive	Cross-domain	MA	1	*Book chapter.*
74	Boddy, A. Enhancing Informed Consent in Oncological Surgery through Digital Platforms and Artificial Intelligence. Clinical Surgical Oncology 2025, 4, 100080, doi:10.1016/j.cson.2025.100080.	Peer-reviewed	Descriptive	Software / digital health	INN, HSI	2	
75	Jian, X.; Wu, B.; Song, Y.; Liu, D.; Wang, Y.; Wang, W.; Zhao, J.; He, D.; Yang, Z.; Zhang, N. Performing Task Automation for Surgical Robot: A Spatial–Temporal Varying Primal–Dual Neural Network wi…	Peer-reviewed	Quantitative-empirical	Surgical instruments/robotics	INN	1	
76	Yin, Q.; Kim, J.; Choi, H.-W.; Kee, S.-J.; Li, R.; Seok, Y.; Bau, H.H. A Self-Heating, Multi-Channel Slider Cassette for Innovative Point-of-Care Molecular Diagnostics from Whole Blood Samples. Bios…	Peer-reviewed	Quantitative-empirical	In vitro diagnostics	INN	1	
77	Salehi, M.; Neo, D.W.K.; Rudel, V.; Stautner, M.; Ganser, P.; Zhang, S.X.; Seet, H.L.; Nai, M.L.S. Digital Manufacturing of Personalized Magnesium Implants through Binder Jet Additive Manufacturing …	Peer-reviewed	Quantitative-empirical	Implantable devices	INN	1	
78	Martjan, R.S.; Weimar, S.N.; Terzidis, O. A Business Model Framework for Software as a Medical Device Startups in the European Union: Mixed Methods Study. Journal of Medical Internet Research 2025, …	Peer-reviewed	Qualitative-empirical	Software / digital health	INN, MA	2	
79	Yadav, S. Europe Surgical Robotics Market Report 2025	Commercial market report	Descriptive	Surgical instruments / robotics	INN, MA	2	
80	Coste, A.T.; Egli, A.; Schrenzel, J.; Nickel, B.; Zbinden, A.; Lienhard, R.; Dumoulin, A.; Risch, M.; Greub, G. IVDR: Analysis of the Social, Economic, and Practical Consequences of the Application …	Peer-reviewed	Quantitative-empirical	In vitro diagnostics	REG, EVI, HSI	3	
81	Semalty, A.; Agrawal, R. Innovation in Knowledge Economy: A Case Study of 3D Printing's Rise in Global Markets and India.	Peer-reviewed	Descriptive	Implantable devices	INN	1	*India, comparator only.*

No.	Reference (abbreviated)	Document type	Evidence type	Device domain	Framework dimension(s)	No. of dimensions	Note
	Recent Advances in Computer Science and Communications 2025, 18, doi:10.2174…						
82	Boretti, A. A Perspective on 3D Printing in the Medical Field. Annals of 3D Printed Medicine 2024, 13, 100138, doi:10.1016/j.stlm.2023.100138.	Peer-reviewed	Descriptive	Implantable devices	INN	1	
83	McKernan, D.; McDermott, O. You Are a Cluster: Now What? The Future of a Medtech Cluster. Journal of Open Innovation: Technology, Market, and Complexity 2024, 10, 100168, doi:10.1016/j.joitmc.2023.1…	Peer-reviewed	Qualitative-empirical	Cross-domain	INN	1	
84	Stern, A.D. Innovation under Regulatory Uncertainty: Evidence from Medical Technology. Journal of Public Economics 2017, 145, 181–200, doi:10.1016/j.jpubeco.2016.11.010.	Peer-reviewed	Quantitative-empirical	Cross-domain	INN	1	*US FDA 1977-2007; non-EU comparator only (Section 9).*
85	Eurostat. Exports of Medicinal and Pharma Products up by 13.5%. 2024	Official statistics	Quantitative-empirical	Cross-domain	MA	1	*Eurostat series covers medicinal and pharmaceutical products*
86	Wu, Y.-H.; Li, F.-A.; Fan, Y.-T.; Tu, P.-W. A Study of Medical Device Regulation Management Model in Asia. Expert Review of Medical Devices 2016, 13, 533–543, doi:10.1080/17434440.2016.1184970.	Peer-reviewed	Legal/policy analysis	Cross-domain	REG	1	*Asia; comparator only.*
87	Eurostat. China-EU International Trade in Goods Statistics	Official statistics	Quantitative-empirical	Cross-domain	MA	1	
88	European Commission. Commission Restricts Chinese Participation in Medical Devices Procurement	Regulatory guidance	Legal/policy analysis	Cross-domain	MA	1	
89	EU Customs Tariff (TARIC) - Taxation and Customs Union	Regulatory guidance	Legal/policy analysis	Cross-domain	MA	1	
90	Dayan, M.; Hervey, T.; McCarey, M.; Fahy, N.; Flear, M.; Greer, S.; Jarman, H. The Future for Health after Brexit.	Research report	Legal/policy analysis	Cross-domain	REG, MA	2	
91	European Court of Auditors. Special Report 27/2023: Screening Foreign Direct Investments in the EU	Regulatory guidance	Legal/policy analysis	Cross-domain	MA	1	*European Court of Auditors special report.*
92	Marley, T.; Barrow, D.; McDermott, O. Global Market and Supply Chain Implications of the European Union Medical Device Regulation 2017/745 as Amended by 2023/607. BMC Health Serv Res 2026, 26, 155, …	Peer-reviewed	Quantitative-empirical	Cross-domain	MA, HSI	2	
93	European Commission, Medical Device Coordination Group Clinical Evaluation of Orphan Medical Devices 2024.	Regulatory guidance	Legal/policy analysis	Cross-domain	REG	1	*MDCG 2024-10.*

Summary of the dimension coding (basis for Table 2 of the manuscript)

Framework dimension	Documents coded	Of which cross-cutting	Note
Regulation	31	15	*Legal / policy analysis*
Evidence	7	6	*Quantitative-empirical (survey)*
Innovation	27	17	*Descriptive / market reporting*
Market Access	41	16	*Descriptive / market reporting*
Healthcare-System Impact	19	12	*Quantitative-empirical (statistics)*
**Documents carrying at least one dimension code**	**91**	**32**	*Column 3 counts documents carrying two or more codes*

Supplementary Material S4 — Screening log: scope, procedure and status (healthcare-4613562)

Manuscript title: The European Medical Device Ecosystem: Interactions Between Regulation, Innovation, Market Access, and Healthcare Systems

1. Purpose of this document

This note explains how the review's evidence base was assembled, the order in which the work was done, and what each supplementary file contains. It accompanies the screening workbook, which holds the record-level data. What follows is the procedure a reader needs in order to judge whether the synthesis rests on a defensible body of evidence.

2. Design and its limits

The review is a structured, protocol-driven synthesis, not a systematic review with meta-analysis. The distinction is substantive rather than presentational. The outcome measures in this literature differ in kind and not merely in magnitude: certification duration in months, development effort as a modelled percentage, market value in currency, adoption and availability as counts and survey proportions, so they cannot be pooled, and no single risk-of-bias instrument spans legislation, official statistics, economic modelling and industry surveys. Identification, screening and reporting nonetheless follow PRISMA 2020 so that the corpus is reproducible and auditable, and the completed checklist records item by item where each requirement is met and where it does not apply.

Reliability is therefore handled at the level of the argument rather than the individual source. Section 11 of the manuscript states six relationships and attaches to each an explicit evidence grade naming the kind of evidence behind it and that evidence's specific weakness. A reader who wants to know how much weight a claim bears should read that grading alongside this log.

3. Order of work

The work was carried out in six stages, in this order. (i) The protocol was fixed: research questions, framework dimensions, eligibility criteria, geographical definition and admissible document types, all reported in Section 2.2 of the manuscript. (ii) Five Boolean search strings were run against four bibliographic databases, and eight institutional and legal sources were searched by hand. (iii) Records were de-duplicated in the reference manager and residual duplicates removed manually. (iv) Titles and abstracts were screened against the inclusion criteria. (v) Full texts of the surviving records were assessed, with every exclusion assigned a reason drawn from the published criteria. (vi) Retained documents were coded on three axes: framework dimension, evidence type and device domain, which produced the distribution reported in Table 2 of the manuscript.

Two features of stage (ii) are worth stating plainly. The institutional sources, e.g., EUR-Lex, the Commission's medical devices and health technology assessment pages, the MDCG guidance repository, EUDAMED documentation, Eurostat, the OECD and MedTech Europe were searched by navigation rather than by query, because the material sought there is legislation, guidance and official statistics that bibliographic databases do not index. For those sources, the 'query' recorded in the workbook is the page or repository consulted, and the count is the number of documents retrieved. And the legal and institutional sources were re-checked at revision, because three developments inside the original window – Implementing Regulation (EU) 2026/977, the Digital Omnibus and the first selection of devices for joint clinical assessment — post-dated the first search and materially affect the argument. This update deviates from the original protocol and is recorded as such in the PRISMA checklist.

4. Screening procedure

Screening was performed independently by two authors at both stages, with disagreements resolved by discussion with the third. Both decisions and the resolution are recorded for every record in the workbook, so that agreement can be recomputed rather than merely asserted. Exclusions at full text carry one of eight reasons: medicinal or combination product assessed as a medicine; single-device clinical study with no regulatory, economic or system-level dimension; outside the EU and EEA and not admitted as an explicit comparator; vendor marketing or commentary without a verifiable data source; duplicate reporting of the same underlying dataset; abstract only retrievable; outside the search period; not published in English. The counts by reason feed the exclusion box of the flow diagram.

Six documents are retained under the comparator provision of the third criterion: they concern the United States, Korea, Asia, or India and are admitted only to establish whether a mechanism observed in the EU is specific to the European framework. They are not counted as EU evidence anywhere in the synthesis. The most important of them is the 1977–2007 United States study of regulatory uncertainty, whose status is stated explicitly in Section 9 and in the grading of the corresponding relationship.

5. Record counts

The counts below are the fields the flow diagram requires. They are to be completed from the workbook; the included total is already fixed by the current reference list.

Stage	Count	Field in S2
Records identified from databases	n = 6226	S2, identification
Records identified from other sources	n = 500	S2, identification
Duplicate records removed	n = 4729	S2, identification
Records screened on title and abstract	n = 1997	S2, screening
Records excluded at title and abstract	n = 1706	S2, screening
Full-text documents assessed	n = 291	S2, screening
Full-text documents excluded, by reason	n = 198	S2, screening
Documents included in the synthesis	93 (current list)	S2, included

6. Coding of the retained corpus

Each retained document was coded on three axes. The first records which of the five framework dimensions it addresses, with multiple codes permitted; a document carrying two or more is counted as cross-cutting. The second records evidence type: quantitative-empirical, qualitative-empirical, modelling or economic analysis, legal or policy analysis, descriptive or market reporting, or method guideline. The third records device domain. The full coded list is S3; the distribution is below.

Framework dimension	Documents coded	Of which cross-cutting	Dominant evidence type
Regulation	30	16	Legal and policy analysis
Evidence	7	5	Quantitative-empirical (one industry survey dominant)
Innovation	27	15	Descriptive technology and market reporting
Market Access	41	19	Descriptive market reporting
Healthcare-System Impact	19	11	Official statistics

7. The supplementary set

S1 is the completed PRISMA 2020 checklist, including the items that do not apply and why. S2 is the flow diagram. S3 is the coded list of included documents, with notes. S4 is the screening workbook, which holds the search-execution record, the de-duplication record, the record-level screening tables for both stages.
